# Synthesis and profiling of a 3-aminopyridin-2-one-based kinase targeted fragment library: Identification of 3-amino-5-(pyridin-4-yl)pyridin-2(*1H*)-one scaffold for monopolar spindle 1 (MPS1) and Aurora kinases inhibition

**DOI:** 10.1016/j.bmc.2018.04.033

**Published:** 2018-07-15

**Authors:** Daren Fearon, Isaac M. Westwood, Rob L.M. van Montfort, Richard Bayliss, Keith Jones, Vassilios Bavetsias

**Affiliations:** a Cancer Research UK Cancer Therapeutics Unit at The Institute of Cancer Research, London SM2 5NG, UK; b Astbury Centre for Structural Molecular Biology, School of Molecular and Cellular Biology, Faculty of Biological Sciences, University of Leeds, UK

**Keywords:** 3-Aminopyridin-2-one, Fragment compound library, Aurora kinase, MPS1 kinase

## Abstract

Screening a 3-aminopyridin-2-one based fragment library against a 26-kinase panel representative of the human kinome identified 3-amino-5-(1-methyl-*1H*-pyrazol-4-yl)pyridin-2(*1H*)-one (**2**) and 3-amino-5-(pyridin-4-yl)pyridin-2(*1H*)-one (**3**) as ligand efficient inhibitors of the mitotic kinase Monopolar Spindle 1 (MPS1) and the Aurora kinase family. These kinases are well recognised as attractive targets for therapeutic intervention for treating cancer. Elucidation of the binding mode of these fragments and their analogues has been carried out by X-ray crystallography. Structural studies have identified key interactions with a conserved lysine residue and have highlighted potential regions of MPS1 which could be targeted to improve activity and selectivity.

## Graphical abstract

Synthesis, biological evaluation, and structural studies of a kinase targeted 3-aminopyridin-2-one-based library identified ligand efficient inhibitors of MPS1 and Aurora kinases.

**Figure:**
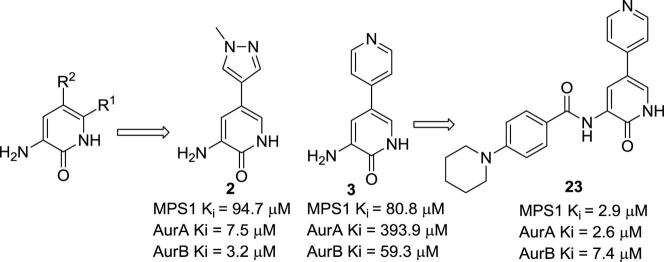

## 1 Introduction

Protein kinases play a key role in protein phosphorylation and cell signal transduction pathways representing an important therapeutic class.^1^, ^2^ Deregulation of protein kinase activity, by over-expression or mutation, is intimately involved in cancer cell proliferation and survival.^1^ At least 28 small-molecule kinase inhibitors have been approved for use in the clinic, the majority of which target part of the highly conserved ATP-binding site, known as the hinge region.^1^, ^3^, ^4^, ^5^, ^6^, ^7^, ^8^ The highly conserved nature of this region presents a common challenge in developing kinase inhibitors, namely obtaining a desirable selectivity profile.^1^, ^9^, ^10^ High throughput screening against a kinase of interest in a target-centric manner is a commonly used approach to identify small-molecule inhibitors. These inhibitors are subsequently screened against a panel of kinases in order to gain an understanding of their selectivity profile. Optimization can then lead to a more potent inhibitor that exhibits a desirable selectivity profile. Despite the successful applications of this method, various limitations have become clear. Several mechanisms by which cancer cells can escape inhibition of a single kinase have been reported.^11^, ^12^, ^13^, ^14^ Additionally, protein kinase drug discovery efforts have focussed on a small number of well-validated targets.^15^ An alternative strategy is to target multiple nodes on a signalling network simultaneously giving the cancer cell a higher evolutionary barrier to overcome in order to achieve resistance.^16^, ^17^ Although the highly conserved active site of protein kinases is often cited as a cause of poor selectivity of kinase inhibitors, it may be possible to exploit this poor selectivity to target multiple kinases.^12^ The recent increase in protein kinase panel screens can allow selectivity profiling of inhibitors to be carried out at a very early stage.^10^, ^18^, ^19^, ^20^, ^21^, ^22^ Screening small-molecule libraries in a target-blind manner against a representative panel of kinases can identify novel chemotypes as inhibitors while giving detailed information of their selectivity profiles. This approach may prove useful in identifying desirable selectivity profiles in order to discover small molecules that target multiple specific kinases that in combination are of clinical significance.

Owing to the successful application of structural biology to the development of small-molecule protein kinase inhibitors from fragments, fragment-based drug design (FBDD) has become an area of sustained interest.^23^, ^24^ Fragment-based hits are generally weak binders but form high quality interactions and can exhibit high ligand efficiency.^25^, ^26^ Fragment compound libraries can sample a greater degree of chemical space than a lead like compound library containing a similar number of small molecules.^23^, ^27^, ^28^, ^29^ Fragments often possess good physicochemical properties and can be subsequently grown or linked to other fragments in order to increase activity and selectivity.^25^, ^26^, ^30^ Despite their low molecular weight, fragments can possess a high degree of selectivity by exploiting very small structural differences.^18^ However, selectivity is not always maintained between a fragment and related lead-like molecules.^18^ The selectivity of small molecules tested against a panel of kinases can be quantitatively described by their selectivity score (S), the ratio of kinases in the panel that are inhibited above a certain% inhibition, e.g. S_compound_(50%).^21^ The score ranges from 0 to 1, with 1 representing a completely non-selective inhibitor. A similar scoring function can be used to describe the selectivity score of a kinase (S_kinase_(50%)). This represents the ratio of compounds in a screening library that inhibit the particular kinase > 50%.^10^

**Fig. 1:**
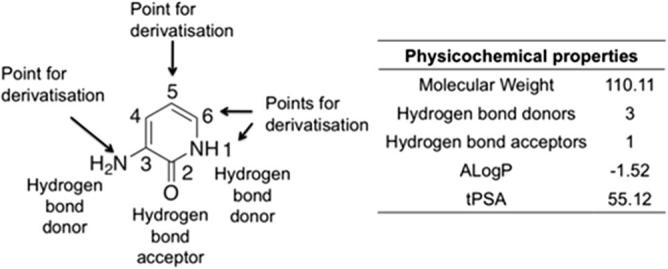
Properties of 3-aminopyridin-2-one fragment **1.**

The identification of novel kinase inhibitor scaffolds is highly desirable in order to develop selective kinase inhibitors. Small-molecule inhibitors of Interleukin-2-inducible *T*-cell kinase (Itk) that are based on the 3-aminopyridin-2-one fragment **1** have been reported.^31^ Despite derivatisation of **1** yielding potent Itk inhibitors,^31^ this 3-aminopyridin-2-one fragment is poorly represented in small-molecule protein kinase modulators. This fragment is capable of forming multiple hydrogen bonds to the backbone of the hinge region, possesses good physicochemical properties^30^ and contains several suitable points for derivatisation (Fig. 1), representing a good starting point for the synthesis of a kinase-targeted fragment library. Herein, we report the synthesis and kinase profiling of a 3-aminopyridin-2-one based fragment library against a panel of 26 protein kinases. This led to the rapid identification of aminopyridin-2-one derivatives as a novel class of inhibitors of monopolar spindle 1 (MPS1) and Aurora kinases, two kinases intimately involved in mitosis.

## 2 Results and discussion

**Fig. 2:**
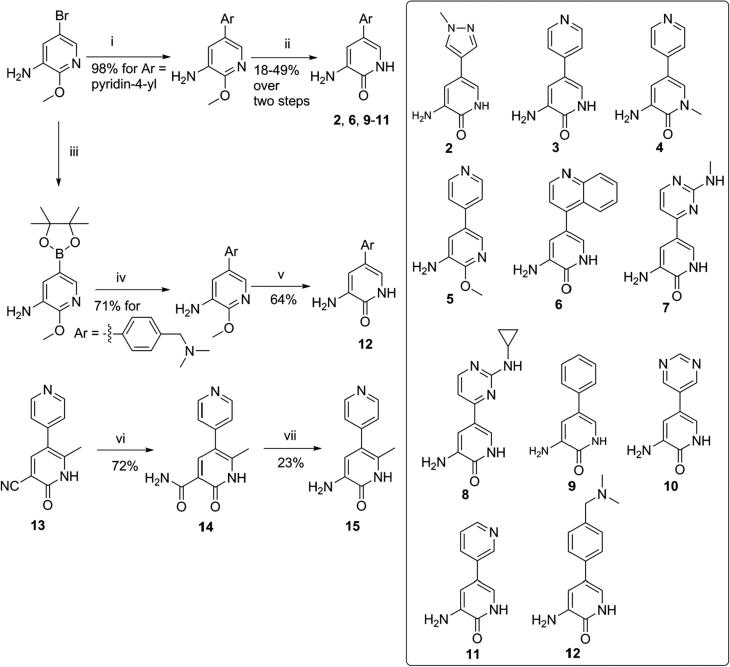
Synthesis and members of 3-aminopyridin-2-one based fragment library. i: Aryl/Heteroaryl boronic acid, Pd_2_(dba)_3_, XPhos, K_3_PO_4_, *n*-butanol, 120 °C. ii: TMS-Cl, NaI, acetonitrile. iii: Bis(pinacolato)diboron, KOAc, Pd(dppf)Cl_2_, dioxane, 80 °C. iv: Aryl/Heteroaryl halide, Pd(PPh_3_)_4_, Na_2_CO_3_, toluene, EtOH, H_2_O, 100 °C. v: TMS-Cl, NaI, acetonitrile. vi: H_2_SO_4_, 120 °C. vii: NaOH, Br_2_. For synthesis of compound **4**, see Supplementary Data Fig. 1, and for the synthesis of compounds **7** and **8**, see Supplementary Data Fig. 2.

A fragment library based on 3-aminopyridin-2-one was synthesised from 5-bromo-2-methoxypyridin-3-amine by first introducing a variety of aromatic and heteroaromatic groups at the 3-aminopyridin-2-one C5-position under standard Suzuki cross-coupling conditions, followed by deprotection of the 3-amino-2-methoxy-pyridine intermediates by generating TMS-I *in situ* (Figure 2).^32^ 3-Amino-6-methyl-5-(pyridin-4-yl)pyridin-2-one (**15**) was synthesised in two steps from the phosphodiesterase inhibitor Milrinone (**13**). Hydrolysis of the nitrile **13** to the carboxamide intermediate **14** by treatment with concentrated sulfuric acid at 120 °C, was followed by a Hofmann rearrangement to give compound **15** (Fig. 2).^33^

**Fig. 3:**
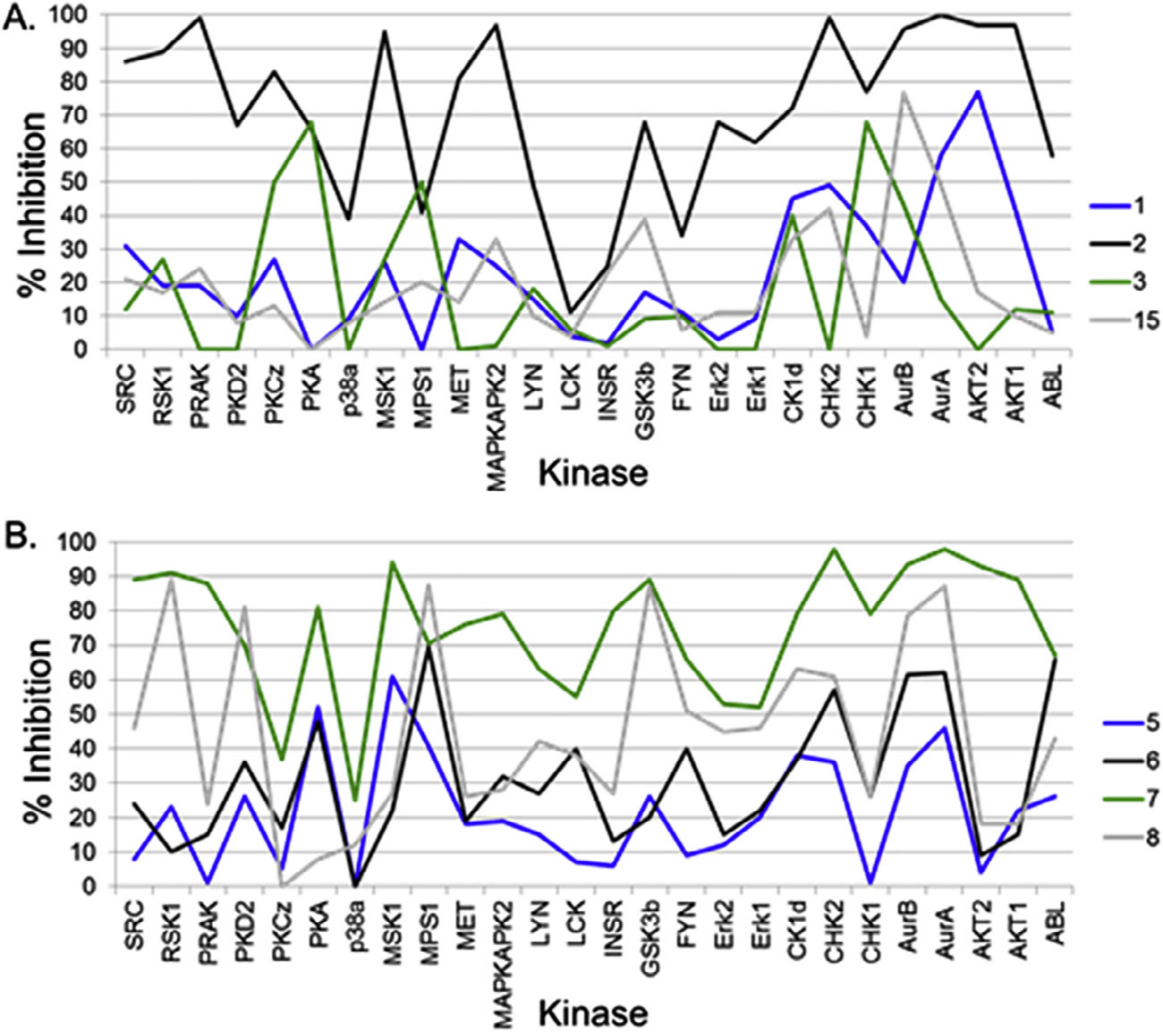
Line chart plotting the% inhibition at 100 µM A: **1** (Blue); **2** (Black); **3** (Green); **15** (Grey); B: **5** (Blue); **6** (Black); **7** (Green); **8** (Grey).

In order to test the biochemical activity of our fragment library, a panel of 26 structurally diverse protein kinases was used. The panel represented members of the TK, CMGC, CAMK, CK1 and AGC families (Supplementary data Table 1). By choosing this panel, we recognised that the exact composition of a kinase panel could affect selectivity scores in particular for small assay panels, as suggested by Karaman et al.^21^ Compounds **1**–**3**, **5**–**8** and **15** were initially screened at a single concentration of 100 µM using a mobility shift-based biochemical assay from Caliper Life Sciences.^34^ The 3-aminopyridin-2-one fragment **1** showed very little activity against our kinase panel with the exception of Aurora A and AKT2, 58% and 77% inhibition at 100 μM, respectively (Fig. 3, and Supplementary data Table 2). This may be expected from screening such a small fragment at a relatively low concentration, as even weak activity such as a Ki of 1 mM would give a ligand efficiency of 0.52. Introduction of an aromatic ring at the C5-position generally resulted in an increase in biochemical activity. Introduction of a *N*-methylpyrazole (compound **2**) gave high percentage inhibition against a large number of kinases in the panel, resulting in a S(50%) of 0.77 (Supplementary data Table 2) showing good potency but poor selectivity. The phosphodiesterase inhibitor Amrinone (**3**), which contains a 4-pyridyl substitution, showed reasonable activity against four members of the panel (MPS1, CHK1, PKCζ and PKA), resulting in a S(50%) score of 0.08. Introduction of a methyl group at the C6-position of the aminopyridinone **3** gave compound **15** which showed low potency against the 26-kinase panel, only one kinase, Aurora B, was inhibited by>50% (77%) (Fig. 3, and Supplementary data Table 2).

This may suggest the methyl group in compound **15** clashes with the protein-binding site, or induces an unfavourable conformation of the biaryl system. Retention of the exocyclic 3-NH_2_ hydrogen bond donor in **3**, accompanying by the removal of the hydrogen bond donor of the pyridone ring by conversion to the 3-amino-2-methoxy-pyridine analogue **5** resulted in a similar inhibitory profile against MPS1 and Aurora B compared to compound **3** (Table 1). An increase in activity against Aurora A, MPS1, ABL and CHK2 was observed when the 4-pyridyl ring in compound **3** was substituted with a quinoline moiety, giving compound **6** (Table 1, Fig. 3). This analogue displayed a selectivity score S(50%) of 0.19 (Supplementary data Table 2). A significant increase in activity across the entire panel was observed by the introduction of a substituted 2-aminopyrimidine moiety. 2-Methylaminopyrimidine analogue **7** gave>50% inhibition against 23 members of our 26-kinase panel (Fig. 3) which resulted in a selectivity score S(50%) of 0.92 (Supplementary data Table 2). Substitution of 2-methylaminopyrimidine in **7** with 2-cyclopropylaminopyrimidine (compound **8**) maintained activity against some members of the panel (AurA, MPS1, GSK3b, RSK1 PKD2) resulting in a selectivity score S(50%) of 0.35 (Supplementary data Table 2). The poor selectivity of the aminopyrimidine analogues **7** and **8** is possibly due to the aminopyrimidine acting as a hinge binding motif as previously reported.^18^, ^35^, ^36^, ^37^, ^38^ In order to validate these single point assay results, the dose-response curves for the compound libraries were obtained against Aurora A, Aurora B and MPS1 kinases. These kinases were selected because of their high S_kinase_(50%) against this compound library (Supplementary data Table 4) and their biological relevance in cancer cell proliferation and survival. The Aurora kinases are well recognised as attractive targets for cancer therapeutics and have extensively been investigated.^39^, ^40^ The inhibition of MPS1 has been also proposed as an effective method for treatment of human cancers, with a relatively limited number of small-molecule MPS1 modulators being reported.^41^, ^42^, ^43^, ^44^, ^45^, ^46^, ^47^

**Table 1:** Ki values and ligand efficiencies for 3-aminopyridin-2-one library against Aurora A, Aurora B and MPS1.

Compd	MPS1 Ki µM (LE)	AurA Ki µM (LE)	AurB Ki µM (LE)
**1**	>1000 (-)	361.0 (0.59)	159.7 (0.64)
**2**	94.7 (0.39)	7.5 (0.53)	3.2 (0.53)
**3**	80.8 (0.40)	393.9 (0.30)	59.3 (0.41)
**4**	236.4 (0.33)	99.5 (0.36)	102.7 (0.36)
**5**	91.2 (0.37)	184.7 (0.31)	63.9 (0.38)
**6**	30.2 (0.34)	76.3 (0.31)	41.7 (0.33)
**7**	25.6 (0.39)	21.3 (0.40)	15.1 (0.41)
**8**	11.0 (0.37)	30.2 (0.34)	12.0 (0.37)
**9**	128.2 (0.38)	66.9 (0.41)	70.3 (0.40)
**10**	269.3 (0.35)	89.6 (0.39)	18.7 (0.46)
**11**	98.1 (0.39)	125.2 (0.38)	97.2 (0.39)
**12**	367.3 (0.26)	>100 (-)	12.9 (0.37)
**13**	>100 (-)	>100 (-)	>100 (-)
**14**	>100 (-)	>100 (-)	>100 (-)
**15**	>100 (-)	>100 (-)	25.7 (0.42)

K_i_ values were calculated from IC_50_ values using the Cheng-Prusoff equation.^48^

IC_50_ values were obtained for an expanded version of the fragment library using the previously described mobility shift assay.^44^ Subsequently, K_i_ values were estimated from IC_50_ values to allow better comparison of the activity against targets which were measured at different substrate concentrations (Supplementary data Table 1). The methylpyrazole analogue **2** showed good activity and high ligand efficiency against both Aurora A and B, with a more modest K_i_ value against MPS1 (Table 1). Compound **3** demonstrated modest inhibition and good ligand efficiency against MPS1 and Aurora B but lower inhibitory activity against Aurora A (Table 1). In agreement with data provided from the single point assay (Fig. 3), compound **15** was only active against Aurora B displaying good ligand efficiency against this kinase (Table 1). The quinoline derivative **6** possessed reasonable activity against all three kinases but a reduction in LE was observed due to the increase in number of heavy atoms (Table 1). Introduction of 2-aminopyrimidine-based substituents (compounds **7**, **8**) resulted in an increase in potency compared to that observed for **3** (Table 1), as it would be expected by the observed trends in percentage inhibition at 100 µM (Fig. 3). Both the methylaminopyrimidine derivative **7** and cyclopropylaminopyrimidine **8** were potent in inhibiting all three kinases, maintaining high ligand efficiency (Table 1). Repositioning of the pyridine nitrogen by substituting the 4-pyridyl with a 3-pyridyl ring, compound **11**, resulted in similar activity to that seen with **3**, and removal of the nitrogen by replacement of the 4-pyridyl in **3** with a phenyl ring, compound **9**, again gave comparable K_i_ values (Table 1). The pyrimidine analogue **10** showed a decrease in activity against MPS1 compared to compound **3**, and a moderate increase in inhibitory activity against Aurora A and B (Table 1). Compound **12** showed selectivity for inhibition of Aurora B over Aurora A and MPS1 (Table 1).

**Fig. 4:**
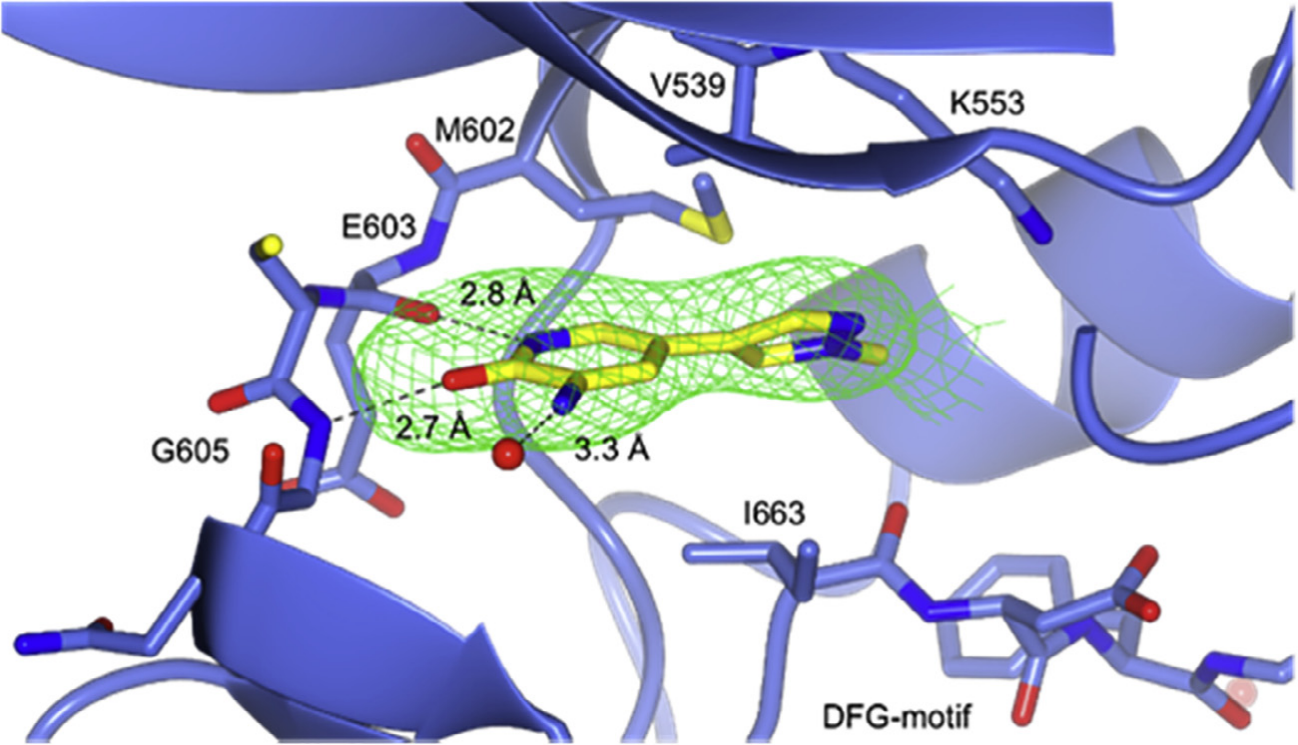
X-ray crystal structure of **2** (carbon atoms in yellow) bound to MPS1 (3.00 Å) with Fo-Fc omit map of **2** contoured at 3*σ*, clipped to 2 Å around **2** and displayed as a green mesh (PDB Code: 4CV8).

The binding mode of these biaryl fragments to MPS1 was elucidated using X-ray crystallography. The crystal structure of the *N*-methylpyrazole analogue **2** in complex with MPS1 was determined with a resolution of 3.00 Å (Fig. 4), and possessed the typical kinase tertiary structure. The positioning of the α C-helix and the DFG motif, and the presence of the catalytic C- and R-spine, suggest that MPS1 is in an active conformation.^44^, ^49^ However, the activation loop (M671 - V684) could not be modelled, indicating that this region is not ordered, and no salt bridge was observed between K553 and E571. Clear electron density for the ligand was observed, identifying key hydrogen bonds formed from the aminopyridinone NH to the carbonyl of E603 (2.8 Å) and from the NH of G605 to the carbonyl of the 3-aminopyridin-2-one (2.7 Å).

**Fig. 5:**
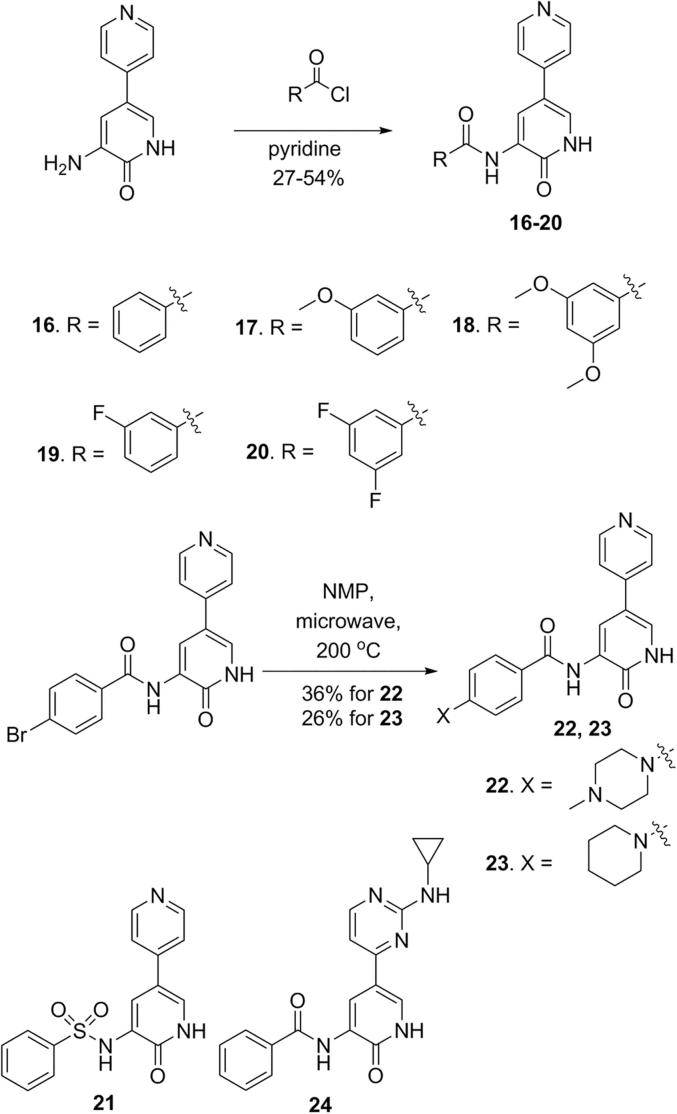
Synthesis of benzamidopyridin-2-one library. For synthesis of compound **24**, see Supplementary Data. Also, for synthesis of compound **21**, see Supplementary Data.

Protein kinases frequently feature a hydrophobic channel that extends from the hinge region to the solvent exposed surface.^50^ Positioning of hydrophobic groups along this channel is a common strategy used to increase activity of kinase inhibitors.^50^ As substitution along this vector grants access to the solvent, it is also exploited to introduce solubilising groups. The amino acid sequence along this channel is less conserved between kinases than the hinge region and can also be exploited to gain selectivity.^50^ The crystal structure of compound **2** bound to MPS1 suggests that this channel is accessible through modification of the 3-amino position (Fig. 4). Owing to this observation and the attributes of the channel that extends from the hinge region, a compound library based on benzamido derivatives of compound **3** was synthesised including a sulfonamido-based derivative, compound **21** (Fig. 5). The amide bond formation reaction and S_N_Ar substitution reactions shown in Fig. 5 have been previously reported for the preparation of compounds of this class.^51^ Compound **3** was selected as it displayed a less promiscuous kinase profile compared with **2**, and it was hypothesised to have a kinase binding mode similar to that of fragment **2**. A selection of members from this library was again screened against a panel of 26 protein kinases.

**Fig. 6:**
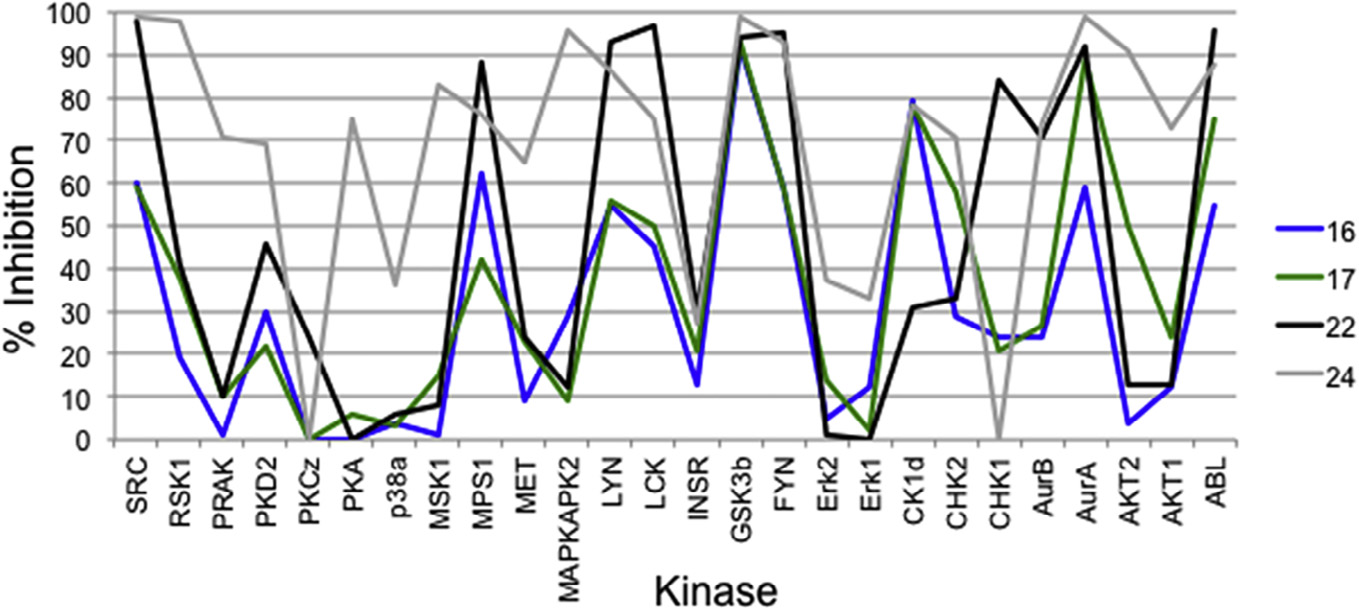
Line chart plotting the% inhibition for compounds **16** (Blue); **17** (Green); **22**: (Black); **24** (Grey).

Screening a selection of analogues from this library against the panel of 26 kinases (**16**, **22**, and **24** were screened at 30 μM due to poor solubility; **17** tested at 100 μM) generally demonstrated an increase in activity compared to compound **3** (Fig. 3, Fig. 6). The benzamide analogue **16** demonstrated >50% inhibition against eight members of the panel including MPS1 and Aurora A (S(50%) = 0.31; Supplementary data Table 3), and the 3-methoxybenzamide analogue **17** also exhibited a selectivity score S(50%) of 0.31 (Fig. 6, Supplementary data Table 3). The *N*-methylpiperazine analogue **22** displayed higher inhibitory potencies compared to **16** while maintaining a selectivity score S(50%) of 0.38 (Fig. 6, Supplementary data Table 3). Analogue **24** showed high percentage inhibition against a large number of kinases in the panel (Fig. 6) with a S(50%) of 0.77 (Supplementary data Table 3). This trend is in agreement with the poor selectivity observed with the structurally related fragments **7** and **8** (Fig. 2, Fig. 3). The decrease in selectivity seen with analogue **24** relative to that observed with **16**, **17**, and **22** may be attributable to the aminopyrimidine system making additional or stronger interactions with the protein or acting as an alternative hinge binding motif. As compounds **16**, **17**, **22**, and **24** often demonstrated > 50% inhibition against Aurora A, Aurora B and MPS1, inhibitory activities against these three kinases were again determined for the expanded compound library (Table 2). The benzamide analogue **16** displayed a 15-fold increase in inhibitory activity against MPS1 relative to that seen with **3,** and also showed significantly higher inhibitory activity against Aurora A compared to that observed for compound **3** (Table 1, Table 2).

**Table 2:** Ki values for benzamidopyridin-2-one library against Aurora A, Aurora B and MPS1.

Compd	MPS1 Ki µM (LE)	AurA Ki µM (LE)	AurB Ki µM (LE)
**16**	5.4 (0.33)	3.3 (0.34)	41.0 (0.27)
**17**	24.8 (0.26)	>100 (-)	>100 (-)
**18**	30.1 (0.24)	>100 (-)	32.9 (0.23)
**19**	>100 (-)	>100 (-)	>100 (-)
**20**	>100 (-)	>100 (-)	>100 (-)
**21**	>100 (-)	>100 (-)	>100 (-)
**22**	4.8 (0.25)	14.9 (0.23)	5.5 (0.25)
**23**	2.9 (0.27)	2.6 (0.27)	7.4 (0.25)
**24**	3.2 (0.29)	17.3 (0.25)	3.6 (0.28)

K_i_ values were calculated from IC_50_ values using the Cheng-Prusoff equation.^48^

Mono- or disubstitution at the *meta*-position of the benzamide **16** with methoxy groups (compounds **17** and **18**) was not well tolerated and equivalent fluorine substitutions (compounds **19** and **20**) led to significant loss of activity (Table 2). The selectivity of compound **17** inhibiting MPS1 over Aurora A and Aurora B could possibly be explained by differences in the main chain conformation along the hydrophobic channel leading to the solvent exposed surface. Introduction of solubilising groups such as *N*-methylpiperazine (compound **22**) and piperidine (compound **23**) retained good activity against all three kinases but resulted in a reduction in ligand efficiency due to the increase in the number of heavy atoms (Table 2). It should be noted that compounds **16** and **23** have been reported by Charrier *et al.* as Itk inhibitors.^31^

**Fig. 7:**
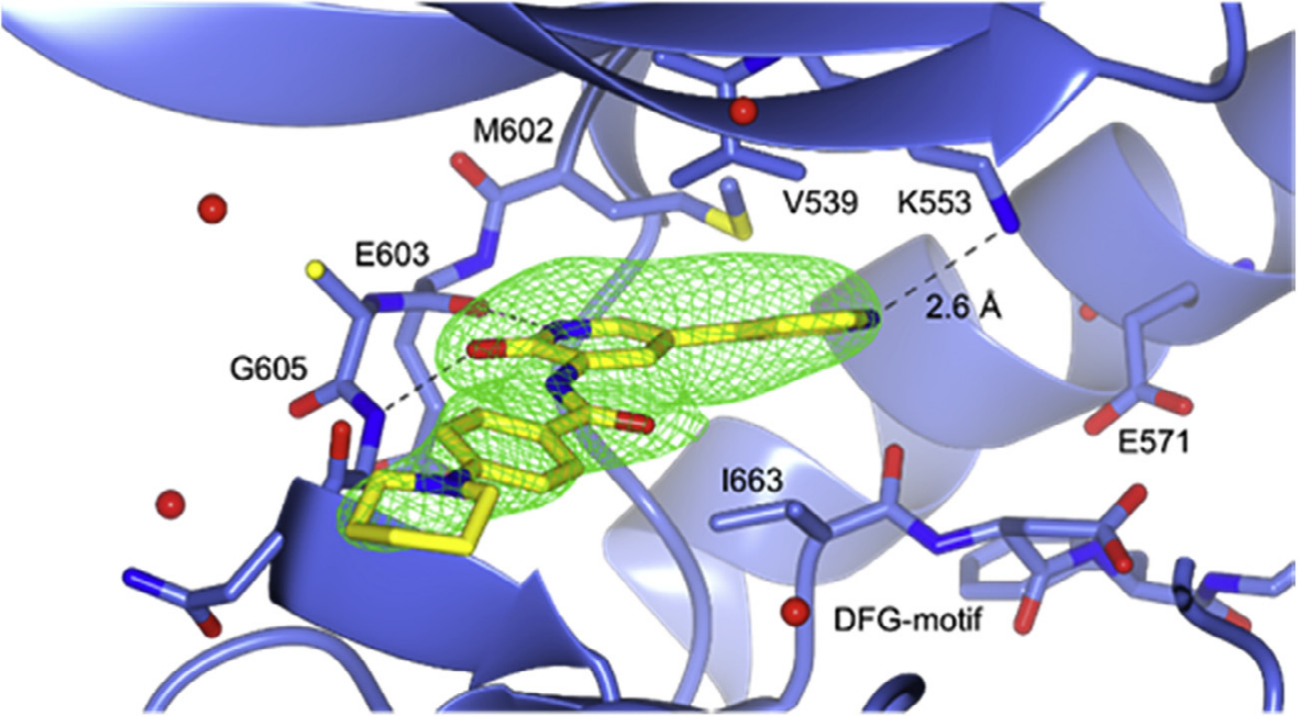
X-ray crystal structure of **23** (carbon atoms in yellow) bound to MPS1 (2.50 Å) with Fo-Fc omit map contoured at 3σ, clipped to 2 Å around **23** and displayed as a green mesh (PDB Code: 4CVA).

**Fig. 8:**
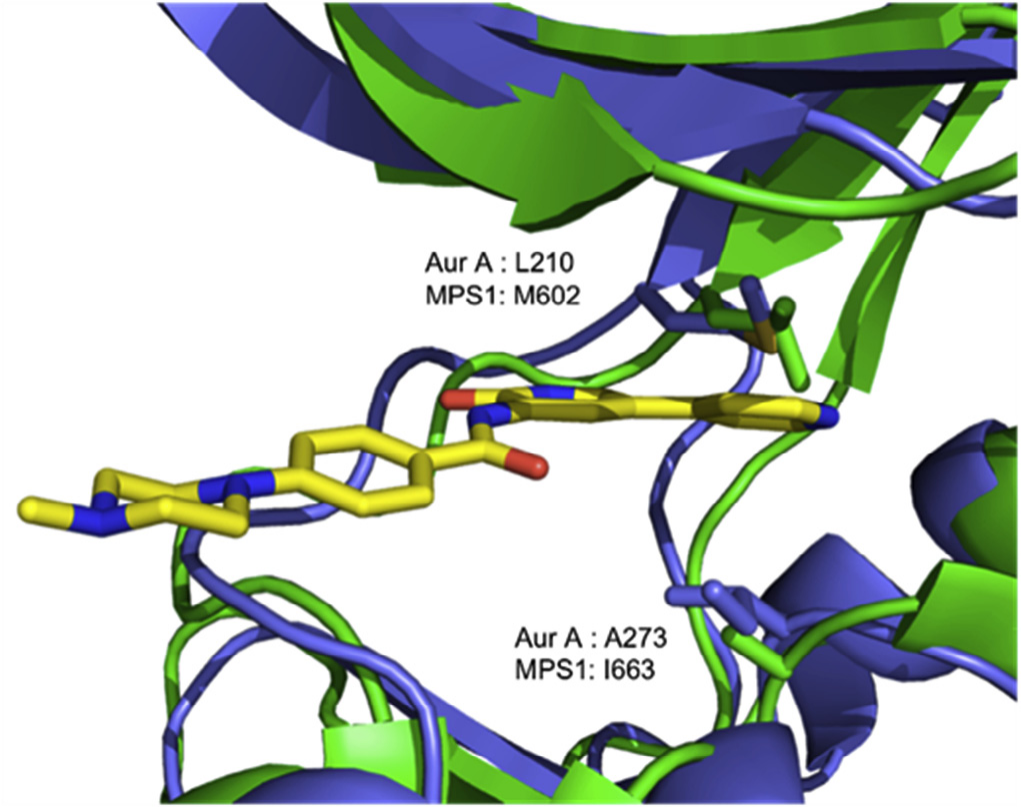
X-ray crystal structure of **22** bound to MPS1 (PDB Code: 4CV9) (purple with I663 and M602 shown as sticks) overlaid with the X-ray crystal structure of Aurora A (green with A273 and L210 shown as sticks).^40^

In order to gain further structural insights into the binding modes of active members of this library, we successfully co-crystallised compounds **22** and **23** with MPS1 (Fig. 7, Fig. 8) with both structures showing a hydrogen bonding pattern to the amino acid backbone of the hinge region identical to that seen with fragment **2** bound to MPS1 (Fig. 4). A further weak hydrogen bond between the nitrogen of the pyridyl ring in **23** and K553 was observed (2.6 Å) as shown in Fig. 7. The piperidine and *N*-methyl piperazine groups present in compounds **23** and **22**, respectively, pointed out towards the solvent exposed surface as hypothesised (Fig. 7, Fig. 8). The pyridyl ring is also involved in hydrophobic packing between V539 and I663 as shown in Fig. 7. Further study of the protein environment of the pyridyl ring system may provide greater understanding of selectivity trends observed across the initial fragment library.

Regarding MPS1 and Aurora kinases, it should be noted that MPS1 possesses a gatekeeper residue which is different to that seen in Aurora A and B kinases. Where MPS1 has a flexible methionine residue (M602), Aurora A and B have a leucine residue (L210 in Aurora A) as shown in Fig. 8. In addition, MPS1 possesses an isoleucine (I663) residue that is involved in hydrophobic packing, the corresponding residue in Aurora A and B is alanine (A273 in Aurora A) (Fig. 8). As previously stated, introduction of a benzamide or substituted benzamide group resulted in a loss in selectivity, both between MPS1 and Aurora A/B, and against the panel of 26 kinases. The hydrophobic channel, into which these groups project, is a common feature observed in kinases. Although the amino acid sequence of the hydrophobic channel is not as highly conserved as that of the hinge region, features of this channel are relatively similar. Since the benzamide analogues **16**, **17**, **22** and **24** show greater percentage inhibition against a large number of the kinases in the panel, it could be assumed that they form non-directional hydrophobic interactions in this region. These findings are in agreement with previous reports suggesting that elaboration of selective fragments does not always result in selective compounds.^18^

## 3 Conclusion

In summary, screening a small compound library based on the 3-aminopyridin-2-one motif in a target-blind manner against a 26-kinase panel, representative of the kinome, identified hits that possess good activity and excellent ligand efficiency against MPS1, Aurora A and Aurora B. The binding mode of fragment **2** and compounds **22** and **23** with MPS1 was elucidated using X-ray crystallography providing structural information that could prove valuable in improving potency and obtaining a desirable selectivity profile. This scaffold may prove an attractive starting point for the development of potent inhibitors of MPS1 by providing vectors suitable for probing the P-loop, as in NMS-P715, or positioning of a hydrophobic group in a manner that further orders the activation loop.^41^, ^44^

## 4 Experimental section

**Chemistry:** All anhydrous solvents and reagents were obtained from commercial suppliers (Sigma Aldrich, Acros, Fluorochem, Alfa Aesar) and used without further purification. Reactions were performed in oven-dried, round-bottom flasks fitted with rubber septa under an atmosphere of argon unless otherwise stated. Analytical thin layer chromatography (TLC) was performed on pre-coated aluminium sheets of silica (60 F_254_, Merck) and visualised by short-wave UV light. Reactions using microwave irradiation were performed in a Biotage® Initiator Sixty. Flash column chromatography was performed on a FlashMaster personal unit using isolute Flash silica columns or a Biotage SP1 purification system using Biotage Flash silica cartridges. Ion exchange chromatography was performed using acidic Isolute Flash SCX-II cartridges. Silicon carbonate columns used were Isolute Si-Carbonate from Biotage.

Semi-Preparative HPLC Method A: 1000 μL standard injections (with needle rinse) of the sample, at 20 mg/mL concentration in DMSO, were made onto a Phenomenex Gemini column (10 μm, 250 × 21.2 mm, C18, Phenomenex, Torrance, USA). Chromatographic separation at room temperature was carried out using Gilson GX-281 liquid handler system combined with a Gilson 322 HPLC pump (Gilson, Middleton, USA) over a 15 min gradient elution from 10:90 to 100:0 methanol:water (both modified with 0.1% formic acid) at a flow rate of 20 mL/min. UV-Vis spectra were acquired at 254 nm on a Gilson 156 UV-Vis detector (Gilson, Middleton, USA). Collection was triggered by UV signal, and collected using a Gilson GX-281 liquid handler system (Gilson, Middleton, USA). Raw data was processed using Gilson Trilution Software.

^1^H NMR spectra were recorded at 500 MHz on a Bruker Avance-500 using an internal deuterium lock. Chemical shifts were measured in parts per million (ppm) relative to tetramethylsilane (*δ* = 0) using the following internal references for residual protons in the solvent: CDCl_3_ (*δ* 7.26), CD_3_OD (*δ* 3.32) and (CD_3_)_2_SO (*δ* 2.50). Data is presented as follows: chemical shift, integration, multiplicity, coupling constant (J) in Hz. ^13^C NMR spectra were recorded at 126 MHz on a Bruker Avance-500 using an internal deuterium lock. Chemical shifts were measured in parts per million (ppm) relative to tetramethylsilane (*δ* = 0) using the following internal references: CDCl_3_ (*δ* 77.0), CD_3_OD (*δ* 49.0) and (CD_3_)_2_SO (*δ* 39.5). Data is presented as follows: chemical shift, integration, multiplicity, coupling constant (J) in Hz.

LC-MS analysis was performed on a Waters LCT with a Waters Alliance 2795 separations module and Waters 2487 dual wavelength absorbance detector coupled to a Waters/Micromass LCT time of flight mass spectrometer with ESI source. Analytical separation was carried out at 30 °C either on a Merck Chromolith SpeedROD column (RP-18e, 50 × 4.6 mm) using a flow rate of 2 mL/min in a 3.5 min gradient elution with detection at 254 nm or on a Merck Purospher STAR column (RP-18e, 30 × 4 mm) using a flow rate of 1.5 mL/min in a 3.5 min gradient elution with detection at 254 nm. The mobile phase was a mixture of methanol (solvent A) and water (solvent B) both containing formic acid at 0.1%. Gradient elution was as follows: 1:9 (A/B) to 9:1 (A/B) over 2.25 min, 9:1 (A/B) for 0.75 min, and then reversion back to 1:9 (A/B) over 0.3 min, finally 1:9 (A/B) for 0.2 min.

HRMS analysis was performed on an Agilent 1200 series HPLC and diode array detector coupled to a 6520 Quadrupole-Time of flight mass spectrometer with dual multimode APCI/ESI source. Analytical separation (Method A) was carried out at 30 °C on a Merck Purospher STAR column (RP-18e, 30 x 4 mm) using a flow rate of 1.5 mL/min in a 4 min gradient elution with detection at 254 nm. The mobile phase was a mixture of methanol (solvent A) and water (solvent B) both containing formic acid at 0.1%. Gradient elution was as follows: 1:9 (A/B) to 9:1 (A/B) over 2.5 min, 9:1 (A/B) for 1 min, and then reversion back to 1:9 (A/B) over 0.3 min, finally 1:9 (A/B) for 0.2 min. The following references masses were used for HRMS analysis: caffeine [M + H]^+^ 195.087652; hexakis(1H,1H,3H-tetrafluoropentoxy)phosphazene [M + H]^+^ 922.009798, and hexakis(2,2-difluoroethoxy) phosphazene [M + H]^+^ 622.02896 or reserpine [M + H]^+^ 609.280657.

### 4.1 3-Amino-5-(1-methyl-*1H*-pyrazol-4-yl)pyridin-2(*1H*)-one (**2)**

5-Bromo-2-methoxypyridin-3-amine (100 mg, 0.493 mmol, 1 equivalent), 1-methyl-1*H*-pyrazol-4-ylboronic acid (74.4 mg, 0.591 mmol, 1.2 equivalents), potassium phosphate (314 mg, 1.478 mmol, 3 equivalents), 2-dicyclohexylphosphino-2,4,6-triisopropylbiphenyl (47 mg, 0.099 mmol, 0.2 equivalents) and tris (dibenzylideneacetone)dipalladium(0) (22.55 mg, 0.025 mmol, 0.05 equivalents) were dissolved in *n*-butanol (4 mL) and stirred at 110 °C for 3 h. The solution was then cooled and filtered through a pad of Celite, which was then washed with methanol. The solution was concentrated and purified by SCX column (eluting at room temperature with 2 M ammonia in methanol) and concentrated. The residue was then dissolved in acetonitrile and sodium iodide (222 mg, 1.484 mmol, 3 equivalents) was added, followed by dropwise addition of trimethylsilyl chloride (0.190 mL, 1.484 mmol, 3 equivalents) and the reaction mixture stirred for 16 h. The solution was concentrated, taken up in methanol and purified by SCX column (eluting at room temperature with 2 M ammonia in methanol) to give a grey solid (25 mg, 27%). R_f_ = 0.36 (5% MeOH in EtOAc). mp: 264 °C. ^1^H NMR (500 MHz, DMSO‑*d*_6_) 3.81 (3H, s), 5.09 (2H, s), 6.63 (1H, d, *J* = 2.3 Hz), 6.83 (1H, d, *J* = 2.3 Hz), 7.59 (1H, s), 7.83 (1H, s), 11.38 (1H, s). ^13^C NMR (126 MHz, DMSO‑*d*_6_) 157.3 (C), 139.2 (C), 135.6 (CH), 127.0 (CH), 119.7 (C), 115.8 (CH), 112.3 (C), 110.1 (CH), 39.0 (CH_3_). HRMS: Found 191.0932, calculated for C_9_H_11_N_4_O (M + H)^+^: 191.0927.

### 4.2 6-Methoxy-3,4′-bipyridin-5-amine (**5)**

#### 4.2.1 General procedure A

2-Dicyclohexylphosphino-2,4,6-triisopropylbiphenyl (141 mg, 0.296 mmol, 0.2 equivalents), tris(dibenzylideneacetone)dipalladium(0) (68 mg, 0.074 mmol, 0.05 equivalents), 4-pyridinylboronic acid (218 mg, 1.773 mmol, 1.2 equivalents) and potassium phosphate (941 mg, 4.43 mmol, 3 equivalents), were added to 5-bromo-2-methoxypyridin-3-amine (300 mg, 1.478 mmol, 1 equivalent) in *n*-butanol (10 mL) and heated to reflux for 3 h. The reaction mixture was then cooled, filtered through a pad of Celite, which was subsequently washed with methanol and concentrated. The residue was then purified using a SCX column, eluting at room temperature with 2 M ammonia in methanol and concentrated to give a brown oil that crystallized upon standing to give a red solid (292 mg, 98%). R_f_ = 0.45 (10% MeOH in CHCl_3_). mp: 204 °C. ^1^H NMR (500 MHz, DMSO‑*d*_6_) 3.92 (3H, s), 5.17 (2H, s), 7.24 (1H, d, *J* = 2.3 Hz), 7.58 (2H, dd, *J* = 1.7 Hz, 4.5 Hz), 7.82 (1H, d, *J* = 2.3 Hz), 8.58 (2H, dd, *J* = 1.6 Hz, 4.5 Hz).^13^C NMR (126 MHz, DMSO‑*d*_6_) 153.1 (C), 150.6 (CH), 145.6 (C), 133.3 (C), 131.2 (CH), 127.5 (C), 121.1 (CH), 116.6 (CH), 53.6 (CH_3_). HRMS: Found 202.0986, calculated for C_11_H_12_N_3_O (M + H)^+^: 202.0975.

#### 4.2.2 2-Methoxy-5-phenylpyridin-3-amine

Prepared using general procedure A to give a brown solid (174 mg, 88%): 2-dicyclohexylphosphino-2,4,6-triisopropylbiphenyl (94 mg, 0.197 mmol, 0.2 equivalents), tris(dibenzylideneacetone)dipalladium(0) (45 mg, 0.049 mmol, 0.05 equivalents), 4-phenylboronic acid (144 mg, 1.182 mmol, 1.2 equivalents), potassium phosphate (627 mg, 2.96 mmol, 3 equivalents), 5-bromo-2-methoxypyridin-3-amine (200 mg, 0.985 mmol, 1 equivalent) and *n*-butanol (7 mL). R_f_ = 0.35 (DCM:EtOAc 1:1). mp: 218 °C. ^1^H NMR (500 MHz, CDCl_3_) 3.87 (2H, s), 4.05 (3H, s), 7.13 (1H, d, *J* = 2.1 Hz), 7.32–7.37 (1H, m), 7.42–7.46 (2H, m), 7.51–7.54 (2H, m), 7.82 (1H, d, *J* = 2.1 Hz).^13^C NMR (126 MHz, CDCl_3_) 152.6 (C), 138.5 (C), 133.3 (CH), 131.0 (C), 130.7 (C), 128.8 (CH), 127.1 (CH), 126.8 (CH), 119.2 (CH), 53.5 (CH_3_). HRMS: Found 201.1030, calculated for C_12_H_13_N_2_O (M + H)^+^: 201.1022.

### 4.3 3-Amino-5-phenyl-pyridin-2-one (**9**)

#### 4.3.1 General procedure B

Trimethylsilyl chloride (0.55 mL, 4.34 mmol, 5 equivalents) was added dropwise to a solution of 2-methoxy-5-phenylpyridin-3-amine (174 mg, 0.869 mmol, 1 equivalent) and sodium iodide (651 mg, 4.34 mmol, 5 equivalents) in acetonitrile (6 mL) and stirred for 2 h at room temperature. The solvent was then removed under reduced pressure and the residue purified using a SCX column to give a grey solid (32 mg, 20%). R_f_ = 0.51 (5% MeOH in CHCl_3_). mp: 291 °C. ^1^H NMR (500 MHz, DMSO‑*d*_6_) 5.16 (2H, s), 6.81 (1H, d, *J* = 2.4 Hz), 6.93 (1H, d, *J* = 2.4 Hz), 7.25 (1H, tt, *J* = 1.2 Hz, 7.3 Hz), 7.36–7.40 (2H, m), 7.44–7.48 (2H, m), 11.53 (1H, br s).^13^C NMR (126 MHz, DMSO‑*d*_6_) 157.5 (C), 139.2 (C), 138.0 (C), 129.3 (CH), 126.9 (CH), 125.7 (CH), 119.6 (C), 117.8 (CH), 110.2 (CH). HRMS: Found 187.0877, calculated for C_11_H_11_N_2_O (M + H)^+^: 187.0866.

#### 4.3.2 2-Methoxy-5-(pyrimidin-5-yl)pyridin-3-amine

Prepared using general procedure A to give a brown solid (142 mg, 72%): 2-dicyclohexylphosphino-2,4,6-triisopropylbiphenyl (94 mg, 0.197 mmol, 0.2 equivalents), tris(dibenzylideneacetone)dipalladium(0) (45 mg, 0.049 mmol, 0.05 equivalents), pyrimidinyl-5-boronic acid (124 mg, 1.182 mmol, 1.2 equivalents), potassium phosphate (627 mg, 2.96 mmol, 3 equivalents), 5-bromo-2-methoxypyridin-3-amine (200 mg, 0.985 mmol, 1 equivalent) and *n*-butanol (7 mL). Rf = 0.3 (DCM: EtOAc 1:1). mp: 233 °C. ^1^H NMR (500 MHz, DMSO‑*d*_6_) 3.92 (3H, s), 5.19 (2H, s), 7.21 (1H, d, *J* = 2.2 Hz), 7.78 (1H, d, *J* = 2.2 Hz), 9.02 (2H, s), 9.16 (1H, s).^13^C NMR (126 MHz, DMSO‑*d*_6_) 157.4 (CH), 154.6 (CH), 152.9 (C), 133.4 (C), 132.0 (C), 131.0 (CH), 124.2 (C), 116.7 (CH), 53.6 (CH_3_). HRMS: Found 203.0929, calculated for C_10_H_11_N_4_O (M + H)^+^: 203.0927.

#### 4.3.3 3-Amino-5-(pyrimidin-5-yl)pyridin-2(1H)-one (**10**)

Prepared using general procedure B to give a brown solid (77 mg, 68%): 2-methoxy-5-(pyrimidin-5-yl)pyridin-3-amine (122 mg, 0.603 mmol, 1 equivalent), trimethylsilyl chloride (0.386 mL, 3.02 mmol, 5 equivalents) and sodium iodide (452 mg, 3.02 mmol, 5 equivalents) in acetonitrile (5 mL). R_f_ = 0.57 (DCM:EtOAc 1:1). mp: 240 °C. ^1^H NMR (500 MHz, DMSO‑*d*_6_) 5.26 (2H, s), 6.82 (1H, d, *J* = 2.4 Hz), 7.20 (1H, d, *J* = 2.4 Hz), 8.94 (2H, s), 9.06 (1H, s).^13^C NMR (126 MHz, DMSO‑*d*_6_) 157.9 (C), 156.7 (CH), 153.7 (CH), 139.6 (C), 131.6 (C), 119.5 (CH), 113.2 (C), 108.6 (CH). HRMS: Found 189.0711, calculated for C_9_H_9_N_4_O (M + H)^+^: 189.0771.

### 4.4 N-(2-Oxo-5-(pyridin-4-yl)-1,2-dihydropyridin-3-yl)benzamide (**16)**

#### 4.4.1 General procedure C

Benzoyl chloride (0.062 mL, 0.53 mmol, 1 equivalent) was added dropwise to 3-amino-5-(pyridin-4-yl)pyridin-2-one (100 mg, 0.53 mmol, 1 equivalent) in pyridine (3 mL) and the mixture was stirred for 16 h at room temperature. The solvent was removed under reduced pressure and the residue taken up in methanol and filtered to give the desired product as a light-yellow solid (41 mg, 27%). R_f_ = 0.58 (1:1 DCM:EtOAc). mp: 291 °C. ^1^H NMR (500 MHz, DMSO‑*d*_6_) 7.54–7.67 (5H, m), 7.82 (1H, d, *J* = 2.5 Hz), 7.90–8.00 (2H, m), 8.52–8.65 (2H, m), 8.77 (1H, d, *J* = 2.5 Hz), 9.39 (1H, s), 12.60 (1H, s).^13^C (126 MHz, DMSO‑*d*_6_) 165.6 (C), 157.8 (C), 150.7 (CH), 144.0 (C), 134.2 (C), 132.7 (CH), 129.5 (C), 129.3 (CH), 128.0 (CH), 127.7 (CH), 122.5 (CH), 120.2 (CH), 115.7 (C). HRMS: Found: 292.1075, calculated for C_17_H_14_N_3_O_2_ (M + H)^+^: 292.1086.

#### 4.4.2 3-Methoxy-N-(2-oxo-5-(pyridin-4-yl)-1,2-dihydropyridin-3-yl)benzamide (**17**)

Prepared using general procedure C to give a yellow solid (23 mg, 27%): 3-methoxybenzoylchloride (0.038 mL, 0.27 mmol, 1 equivalent), 3-amino-5-(pyridin-4-yl)pyridin-2-one (50 mg, 0.27 mmol, 1 equivalent). R_f_ = 0.69 (DCM:EtOAc 1:1). mp: 287 °C. ^1^H NMR (500 MHz, DMSO‑*d*_6_) 3.85 (3H, s), 7.21 (1H, dd, *J* = 1.7 Hz, 8.0 Hz), 7.43–7.60 (3H, m), 8.17–8.31 (3H, m), 8.79–8.88 (3H, m), 9.46 (1H, s), 13.01 (1H, s).^13^C NMR (126 MHz, DMSO‑*d*_6_) 165.6 (C), 159.9 (C), 158.1 (C), 152.3 (C), 142.8 (CH), 135.6 (C), 132.3 (CH), 130.5 (CH), 129.6 (C), 122.6 (CH), 122.0 (CH), 119.8 (CH), 118.5 (CH), 113.1 (CH), 55.9 (CH_3_). HRMS: Found 322.1182, calculated for C_18_H_16_N_3_O_3_ (M + H)^+^: 322.1186.

#### 4.4.3 3,5-Dimethoxy-N-(2-oxo-5-(pyridin-4-yl)-1,2-dihydropyridin-3-yl)benzamide (**18**)

Prepared using general procedure C to give a grey solid (32 mg, 34%): 3,5-dimethoxybenzoylchloride (53.6 mg, 0.27 mmol, 1 equivalent), 3-amino-5-(pyridin-4-yl)pyridin-2-one (50 mg, 0.27 mmol, 1 equivalent). R_f_ = 0.67 (DCM:EtOAc 1:1). mp: 280 °C. ^1^H NMR (500 MHz, DMSO‑*d*_6_) 3.83 (6H, s), 6.75 (1H, t, *J* = 2.2 Hz), 7.06 (2H, d, *J* = 2.2 Hz), 7.61 (2H, dd, *J* = 4.6 Hz, 1.5 Hz), 7.83 (1H, d, *J* = 2.5 Hz), 8.59 (2H, dd, *J* = 4.6 Hz, 1.5 Hz), 8.70 (1H, d, *J* = 2.5 Hz), 9.36 (1H, s), 12.58 (1H, s).^13^C NMR (126 MHz, DMSO‑*d*_6_) 165.4 (C), 161.1 (C), 157.9 (C), 150.7 (CH), 144.0 (C), 136.5 (C), 129.6 (C), 128.4 (CH), 123.3 (CH), 120.1 (CH), 115.5 (C), 105.6 (CH), 104.3 (CH), 56.0 (CH_3_). HRMS: Found 352.1291, calculated for C_19_H_18_N_3_O_4_ (M + H)^+^: 352.1292.

#### 4.4.4 3-Fluoro-N-(2-oxo-5-(pyridin-4-yl)-1,2-dihydropyridin-3-yl)benzamide (**19**)

Prepared using general procedure C to give a yellow solid (24 mg, 29%): 3-fluorobenzoylchloride (0.028 mL, 0.27 mmol, 1 equivalent), 3-amino-5-(pyridin-4-yl)pyridin-2-one (50 mg, 0.27 mmol, 1 equivalent). R_f_ = 0.51 (DCM:EtOAc 1:1). mp: 292 °C. ^1^H NMR (500 MHz, DMSO‑*d*_6_) 7.50 (1H, td, *J* = 2.1 Hz, 8.2 Hz), 7.62 (1H, t, *J* = 8.0 Hz), 7.77 (1H, d, *J* = 2.0 Hz), 7.82 (1H, d, *J* = 7.8 Hz), 8.23 (2H, d, *J* = 6.8 Hz), 8.26 (1H, brs), 8.82 (1H, d, *J* = 2.6 Hz), 8.83 (2H, d, *J* = 6.8 Hz), 9.62 (1H, s), 13.01 (1H, s). ^13^C (126 MHz, DMSO‑*d*_6_) 164.8 (C), 162.5 (d, *J* = 244.8 Hz, CF), 158.1 (C), 152.2 (CH), 142.8 (C), 136.5 (d, *J* = 6.9 Hz, C), 132.7 (CH), 131.5 (d, *J* = 8.0 Hz, CH), 129.5 (C), 124.0 (d, *J* = 1.9 Hz, CH), 123.5 (CH), 121.9 (CH), 119.6 (d, *J* = 21.2 Hz, CH), 114.9 (d, *J* = 23.0 Hz, CH), 113.0 (C). HRMS: Found 310.0984, calculated for C_17_H_13_N_3_O_2_F (M + H)^+^: 310.0986.

#### 4.4.5 Kinase profiling

IC_50_ values for MPS1, Aurora A and Aurora B inhibition were determined as previously described,^44^ and K_i_ values were estimated from the mean IC_50_ values using the Cheng-Prusoff equation.^48^ The protocol for screening against the 26-kinase panel using a mobility shift assay is provided in the Supplementary Material.

#### 4.4.6 Crystallography

Co-crystals of **22** and **23** with MPS1 were produced by vapour diffusion using the hanging-drop method. The well buffer contained 0.2 M NaCO_2_H, 0.1 M BTP (pH 7.5) and 15% (w/v) PEG 3350. The protein solution contained 11.59 mg/mL MPS1, 50 mM HEPES (pH 7.5), 150 mM NaCl and 5 mM DTT, supplemented with 1 mM compound (1% DMSO). Drops contained a 1:1 mixture of well buffer and protein solution. Crystals grew over 3 days at 18 °C. Harvested crystals were briefly transferred to cryoprotectant (22.5% ethylene glycol, 15.5% (w/v) PEG 3350 and 0.08 M Bis-Tris propane pH 7.5) and flash cooled in liquid nitrogen. Crystals of MPS1 in complex with **2** were obtained by back-soaking a crystal of MPS1 with **23** in a new solution of 0.19 M NaCO_2_H, 0.095 M BTP (pH 7.5), 14.25% PEG 3350 supplemented with 2 mM **2** for 48 h prior to harvesting. Structure solution and refinement data are presented in supplementary material.
