# Supplementary material for: Synthesis and profiling of a 3-aminopyridin-2-one-based kinase targeted fragment library: Identification of 3-amino-5-(pyridin-4-yl)pyridin-2(1H)-one scaffold for monopolar spindle 1 (MPS1) and Aurora kinases inhibition
Source: Bioorg Med Chem. 2018 Jul 15;26(11):3021–9. doi: 10.1016/j.bmc.2018.04.033 (PMC6008489; doi:10.1016/j.bmc.2018.04.033)
Supplement: Supplementary data 1 [file mmc1.pdf]

Synthesis and profiling of a 3-aminopyridin-2-one-based kinase targeted fragment library: Identification of 3-amino-5-(pyridin-4-yl)pyridin-2(*1H*)-one scaffold for monopolar spindle 1 (MPS1) and Aurora kinases inhibition

Daren Fearon[a], Isaac M. Westwood[a], Rob L. M. van Montfort[a], Richard Bayliss[b], Keith Jones[a,*], Vassilios Bavetsias[a,*]

[a]Cancer Research UK Cancer Therapeutics Unit at The Institute of Cancer Research, London SM2 5NG, UK.

[b]Astbury Centre for Structural Molecular Biology, School of Molecular and Cellular Biology, Faculty of Biological Sciences, University of Leeds.

*Corresponding authors. E-mail addresses: Keith.Jones@icr.ac.uk (for KJ) and Vassilios.Bavetsias@icr.ac.uk (for VB).

Pages 02-12: Experimental procedures for compound synthesis
Pages 13-18: Biochemical screening and assay protocols
Pages 19-20: Crystallography, structure solution and refinement
Pages 21-37: Copies of ^{1}H NMRs
Pages 38-39: References

Synthesis

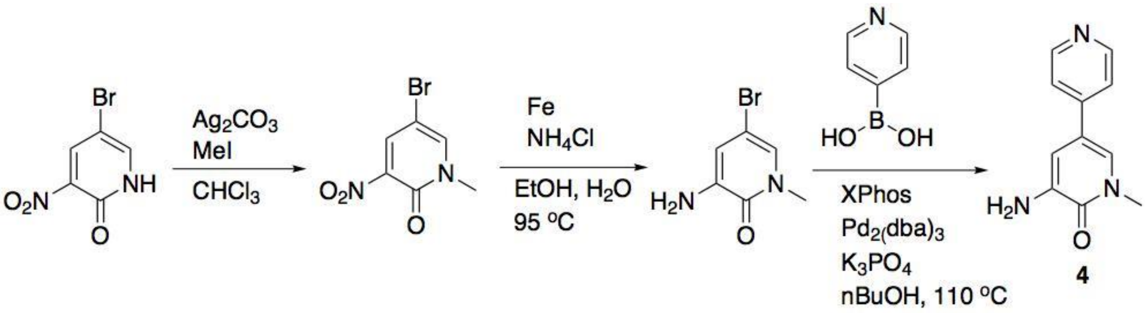

Figure 1: Synthesis of 1-methyl-3-amino-5-(pyridin-5-yl)pyridin-2-one **4**

1-Methyl-3-nitro-5-bromopyridin-2-one

Silver carbonate (4.09 g, 14.84 mmol, 1.3 equivalents) and methyl iodide (7.11 mL, 114 mmol, 10 equivalents) were added to a covered flask containing 5-bromo-3-nitropyridin-2-one (2.5 g, 11.42 mmol, 1 equivalent) and $CHCl_3$ (40 mL) and the reaction allowed to stir at ambient temperature for 2 days. The solution was then filtered through a pad of celite (5 g) and concentrated, giving a yellow solid which was purified by column chromatography (20% EtOAc in hexane, followed by DCM:EtOAc 1:1) to give 1.11 g (42%) of 1-methyl-3-nitro-5-bromopyridin-2-one as an orange solid. R_f = 0.33 (DCM:EtOAc 1:1). mp: 124 °C. ^1H NMR (500 MHz, $CDCl_3$) 3.70 (3H, s), 7.84 (1H, d, J = 2.8 Hz), 8.39 (1H, d, J = 2.8 Hz). ^{13}C (126 MHz, $CDCl_3$) 153.4 (C), 144.6 (CH), 141.2 (CH), 99.9 (C), 94.1 (C), 39.0 (CH_3). HRMS: Found 232.9558 and 234.9539, calculated for $C_6H_6{}^{79}BrN_2O_3$ $(M+H)^+$: 232.9556, calculated for $C_6H_6{}^{81}BrN_2O_3$ $(M+H)^+$: 234.9536. ν_{max} cm^{-1} 1344 (NO_2), 1594 (C=O).

1-Methyl-3-amino-5-bromopyridin-2-one

Iron powder (1 g, 18 mmol, 4.2 equivalents) and ammonium chloride (0.964 g, 18 mmol, 4.2 equivalents) were added to a solution of 1-methyl-3-nitro-5-bromo-pyridin-2-one (1 g, 4.3 mmol, 1 equivalent) in ethanol (8 mL) and water (6 mL) and stirred at 95 °C for 3 hours. The solution was then filtered through a pad of celite and concentrated. The residue was then taken up in water (20 mL) and extracted with EtOAc (3 x 30 mL). The organic layers were dried over magnesium sulfate and concentrated to give a brown oil that solidified upon standing. The residue was purified by SCX to give the desired product as a dark red solid (300 mg, 34%). R_f = 0.5 (DCM:EtOAc 1:1). mp: 200 °C. ^1H NMR (500 MHz, $CDCl_3$) 3.55 (3H, s), 4.37 (2H, s), 6.57 (1H, d, J = 2.4 Hz), 6.83 (1H,

d, J = 2.4 Hz).^{13}C NMR (126 MHz, DMSO-d_6) 156.5 (C), 139.8 (C), 124.2 (CH), 111.9 (CH), 98.7 (C), 37.3 (CH_3). HRMS: Found 202.9815 and 204.9794, calculated for $C_6H_8{}^{79}BrN_2O$ $(M+H)^+$: 202.9815, calculated for $C_6H_8{}^{81}BrN_2O$ $(M+H)^+$: 204.9794. ν_{max} cm^{-1} 3314 (NH_2), 1568 (C=O).

1-Methyl-3-amino-5-(pyridin-5-yl)pyridin-2-one (**4**)

4-Pyridyl boronic acid (73 mg, 0.591 mmol, 1.2 equivalents), 2-dicyclohexylphosphino-2,4,6-triisopropylbiphenyl (47 mg, 0.99 mmol, 0.2 equivalents), potassium phosphate (315 mg, 1.478 mmol, 3 equivalents) and tris(dibenzylideneacetone) dipalladium(0) (23 mg, 0.025 mmol, 0.05 equivalents) were added to 1-methyl-3-amino-5-bromopyridin-2-one (100 mg, 0.493 mmol, 1 equivalent) in butan-1-ol (5 mL) and the reaction mixture was heated to 110 °C for 3 hours. The solution was then cooled and filtered through a pad of celite, which was washed with MeOH. The solution was concentrated and the residue taken up in MeOH and filtered through a SCX column and then purified by column chromatography (5% MeOH in $CHCl_3$) to give a brown solid (45 mg, 46%). R_f = 0.67 (DCM:EtOAc 1:1). mp: 236 °C. ^{1}H NMR (500 MHz, DMSO-d_6) 3.53 (3H, s), 5.31 (2H, s), 6.87 (1H, d, J = 2.5 Hz), 7.50 (2H, dd, J = 1.5 Hz, 4.7 Hz), 7.62 (1H, d, J = 2.5 Hz), 8.54 (2H, dd, J = 1.5 Hz, 4.7 Hz).^{13}C NMR (126 MHz, DMSO-d_6) 157.5 (C), 150.5 (CH), 144.9 (C), 138.8 (C), 124.9 (CH), 119.9 (CH), 116.0 (C), 107.6 (CH), 37.6 (CH_3). HRMS: Found 202.0975, calculated for $C_{11}H_{12}N_3O$ $(M+H)^+$: 202.0975.

3-Amino-5-(quinolin-4-yl)pyridin-2(1H)-one (**6**)

5-Bromo-2-methoxypyridin-3-amine (100 mg, 0.49 mmol, 1 equivalent), quinoline-4-boronic acid (102 mg, 0.59 mmol, 1.2 equivalents), potassium phosphate (314 mg, 1.48 mmol, 3 equivalents), 2-dicyclohexylphosphino-2,4,6-triisopropylbiphenyl (47 mg, 0.10 mmol, 0.2 equivalents) and tris(dibenzylideneacetone)dipalladium(0) (22.55 mg, 0.025 mmol, 0.05 equivalents) were dissolved in *n*-butanol (4 mL) and stirred at 110 °C for 3 hours. The solution was then cooled and filtered through a pad of celite, which was then washed with methanol. The solution was concentrated and purified by SCX column (eluting at room temperature with 2 M ammonia in methanol) and concentrated. The residue was then dissolved in acetonitrile and sodium iodide (222 mg, 1.484 mmol, 3 equivalents) was added followed by dropwise addition of trimethylsilyl chloride (0.190 mL, 1.484 mmol, 3 equivalents) and the reaction mixture stirred for 16 hours. The solution was concentrated, taken up in methanol and purified

by SCX column (eluting at room temperature with 2 M ammonia in methanol) followed by column chromatography (5% MeOH in EtOAc) to give the product as a grey solid (33 mg, 24%). R_f = 0.31 (5% MeOH in EtOAc). mp: 191 °C. ^{1}H NMR (500 MHz, DMSO-d_6) 5.30 (2H, s), 6.66 (1H, d, J = 2.3 Hz), 6.86 (1H, d, J = 2.3 Hz), 7.40 (1H, d, J = 4.4 Hz), 7.62 (1H, ddd, J = 1.4 Hz, 6.8 Hz, 8.4 Hz), 7.78 (1H, ddd, J = 1.4 Hz, 6.8 Hz, 8.4 Hz), 8.00 - 8.15 (2H, m), 8.87 (1H, d, J = 4.4 Hz), 11.72 (1H, s).^{13}C NMR (126 MHz, DMSO-d_6) 157.6 (C), 150.7 (CH), 148.7 (C), 145.1 (C), 139.1 (C), 130.0 (CH), 129.8 (CH), 127.2 (CH), 126.6 (C), 125.9 (CH), 121.4 (CH), 120.7 (CH), 116.7 (C), 112.1 (CH). HRMS: Found 238.0980, calculated for $C_{14}H_{12}N_3O$ $(M+H)^+$: 238.0975.

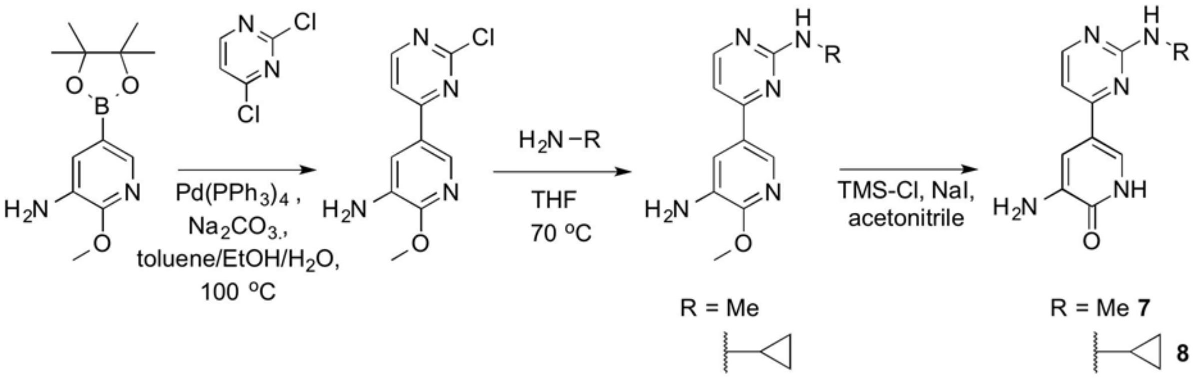

Figure 2: Synthesis of 3-amino-5-(2-(methylamino)pyrimidin-4-yl)pyridin-2(1H)-one **7** and 3-amino-5-(2-(cyclopropylamino)pyrimidin-4-yl)pyridin-2(1H)-one **8**

2-Methoxy-3-amino-5-(4,4,5,5-tetramethyl-1,3,2-dioxaborolan-2-yl)pyridine

5-Bromo-2-methoxypyridin-3-amine (2.0 g, 9.85 mmol, 1 equivalent) was added to dioxane followed by bis(pinacolato)diboron (5.0 g, 19.7 mmol, 2 equivalents), potassium acetate (2.9 g, 29.6 mmol, 3 equivalents) and (1,1'-bis(diphenylphosphosphinol)ferrocene)dichloropalladium(II) dichloromethane adduct (0.804 g, 0.985 mmol, 0.1 equivalents) and stirred at 80 °C for 16 hours under argon. After cooling the solution was diluted with water and the product extracted with EtOAc. The combined organic layers were washed with brine, dried over magnesium sulfate and concentrated. The product was then purified by column chromotography (DCM:EtOAc 1:1) and concentrated. The residue was triturated with diethyl ether to give the product as a yellow solid (950 mg, 39%). R_f = 0.73 (DCM:EtOAc 1:1).

mp: 137 °C. ^1H NMR (500 MHz, DMSO-d_6) 1.27 (12H, s), 3.87 (3H, s), 4.92 (2H, s), 7.12 (1H, d, J = 1.7 Hz), 7.64 (1H, d, J = 1.7 Hz).^{13}C NMR (126 MHz, DMSO-d_6) 154.6 (C), 139.9 (CH), 132.4 (C), 124.2 (CH), 83.9 (C), 53.4 (CH_3), 25.1 (CH_3). HRMS: Found 251.1566, calculated for $C_{12}H_{20}BN_2O_3$ $(M+H)^+$: 251.1564.

2-Methoxy-3-amino-5-((2-chloro)pyrimidin-4-yl)pyridine

2-Methoxy-3-amino-5-(4,4,5,5-tetramethyl-1,3,2-dioxaborolan-2-yl)pyridine (500 mg, 2.0 mmol, 1 equivalent) was dissolved in toluene (20 mL) and ethanol (5 mL) and then 2,4-dichloropyrimidine (357 mg, 2.40 mmol, 1.2 equivalents) was added followed by tetrakis(triphenylphosphine)palladium(0) (116 mg, 0.1 mmol, 0.05 equivalents). Sodium carbonate (1.46 g, 13.79 mmol, 6.9 equivalents) and water (7.5 mL) were then added and the reaction mixture heated to reflux for 2 hours under argon. After cooling the solution was diluted with 15 mL water and extracted with EtOAc (3x50 mL) and the organic layers collected and washed with brine. The organic layers were dried over magnesium sulfate and concentrated. The residue was purified using an SCX column, eluting at room temperature with 2 M ammo-nia in methanol, and triturated with diethyl ether to give the desired product as a grey solid (413 mg, 73%). R_f = 0.47 (5% MeOH in EtOAc). ^1H NMR (500 MHz, DMSO-d_6) 3.95 (3H, s), 5.32 (2H, s), 7.65 (1H, d, J = 1.8 Hz), 8.02 (1H, d, J = 5.3 Hz), 8.24 (1H, d, J = 1.8 Hz), 8.72 (1H, d, J = 5.3 Hz). HRMS: Found 238.0563, calculated for $C_{10}H_{10}ClN_4O$ $(M+H)^+$: 238.0564.

2-Methoxy-3-amino-5-((2-methylamino)pyrimidin-4-yl)pyridine

2-Methoxy-3-amino-5-((2-chloro)pyrimidin-4-yl)pyridine (80 mg, 0.338 mmol, 1 equivalent) was added to 2 M methylamine in THF (3.4 mL) and the reaction mixture stirred in a sealed tube at 70 °C for 24 hours. The solvent was then removed under reduced pressure and the product purified by SCX column (eluting at room temperature with 2 M ammonia in methanol) to give the desired product as a pale yellow solid (50 mg, 64%). R_f = 0.47 (1:1 DCM: EtOAc). ^1H NMR (500 MHz, $CDCl_3$) 3.07 (3H, d, J = 5.2 Hz), 3.91 (2H, brs), 4.06 (3H, s), 5.15 (1H, d, J = 5.2 Hz), 6.90 (1H, d, J = 5.2 Hz), 7.61 (1H, s), 8.23 (1H, s), 8.31 (1H, d, J = 5.2 Hz).^{13}C NMR (126 MHz, $CDCl_3$) 162.9 (C), 158.2 (CH), 149.8 (C), 134.6 (CH), 123.9 (C), 121.6 (C), 118.1 (CH), 105.7 (CH), 100.0 (C), 53.3 (CH_3), 28.3 (CH_3). HRMS: Found 232.1190, calculated for $C_{11}H_{14}N_5O$ $(M+H)^+$: 232.1198.

*3-Amino-5-(2-(methylamino)pyrimidin-4-yl)pyridin-2(1H)-one (**7**)*

Methylamine in THF (2 M, 5 mL) was added to 2-methoxy-3-amino-5-((2-chloro) pyrimidin-4-yl)pyridine (80 mg, 0.34 mmol, 1 equivalent) and stirred overnight at 70 °C. The solvent was then removed and the residue taken up in methanol and purified by SCX (eluted with 2 M ammonia in methanol). The residue was then taken up in acetonitrile (2 mL) and sodium iodide (162 mg, 1.08 mmol, 3.2 equivalents) was added followed by dropwise addition of trimethylsilyl chloride (0.138 mL, 1.08 mmol, 3.2 equivalents). After stirring for 2 hours the solvent was removed and the residue purified by semiprep HPLC (Method A) to give the product as a pale yellow solid (12 mg, 16%). R_f = 0.80 (10% MeOH in $CHCl_3$). mp: 278 °C. ^1H NMR (500 MHz, DMSO-d_6) 2.83 (3H, d, J = 4.4 Hz), 5.19 (2H, s), 6.84 (1H, d, J = 4.6 Hz), 6.93 (1H, d, J = 4.5 Hz), 7.16 (1H, s), 7.52 (1H, s), 8.19 (1H, d, J = 4.6 Hz), 11.53 (1H, brs).^{13}C NMR (126 MHz, DMSO-d_6) 186.6 (C), 163.1 (C), 158.6 (CH), 158.4 (C), 138.7 (C), 121.6 (CH), 117.1 (C), 108.3 (CH), 100.6 (CH), 28.4 (CH_3). HRMS: Found 218.1037, calculated for $C_{10}H_{12}N_5O$ $(M+H)^+$: 218.1036.

2-Methoxy-3-amino-5-((2-cyclopropylamino)pyrimidin-4-yl)pyridine

Cyclopropylamine (0.7 mL, 10.1 mmol, 20 equivalents) was added to 2-methoxy-3-amino-5-((2-chloro)pyrimidin-4-yl)pyridine (120 mg, 0.51 mmol, 1 equivalent) in THF (3.5 mL) and stirred at 70 °C for 16 hours. The solvent was then removed under reduced pressure to give an orange oil that was purified by SCX column (eluted with 2 M ammonia in methanol) to give a pale yellow solid (60 mg, 46%) R_f =0.47 (DCM:EtOAc 1:1). mp: 230 °C. ^1H NMR (500 MHz, DMSO-d_6) 0.45 - 0.52 (2H, m), 0.63 - 0.75 (2H, m), 2.77 (1H, m), 5.13 (2H, s), 7.02 (1H, d, J = 5.2 Hz), 7.31 (1H, d, J = 3.5 Hz), 7.55 (1H, brs), 8.11 (1H, brs), 8.29 (1H, d, J = 5.0 Hz).^{13}C NMR (126 MHz, DMSO-d_6) 163.9 (C), 162.5 (C), 158.8 (CH), 154.0 (C), 132.8 (C), 132.4 (CH), 127.4 (C), 116.8 (CH), 106.0 (CH), 53.7 (CH_3), 24.4 (CH), 6.9 (CH_2). HRMS: Found 259.1381, calculated for $C_{13}H_{16}N_5O$ $(M+H)^+$: 259.1376.

*3-Amino-5-(2-(cyclopropylamino)pyrimidin-4-yl)pyridin-2(1H)-one (**8**)*

Trimethylsilyl chloride (0.15 mL, 1.17 mmol, 5 equivalents) was added dropwise to a solution of 2-methoxy-3-amino-5-((2-cyclopropylamino)pyrimidin-4-yl)pyridine (60 mg, 0.23 mmol, 1 equivalent) and sodium iodide (175 mg, 1.17 mmol, 5 equiv-alents) in acetonitrile (2.5 mL) and stirred for 2 hours at room temperature. The solvent was

then removed under reduced pressure and the product purified by semiprep HPLC (Method A) to give a pale yellow solid (28 mg, 49%). R_f = 0.51 (10% MeOH in $CHCl_3$). mp: 218 °C. ^1H NMR (500 MHz, DMSO-d_6) 0.48 (2H, m), 0.67 (2H, m), 2.70 - 2.78 (1H, m), 5.19 (2H, s), 6.89 (1H, d, J = 5.3 Hz), 7.16 (1H, s), 7.22 (1H, d, J = 3.5 Hz), 7.53 (1H, s), 8.22 (1H, d, J = 5.3 Hz), 11.73 (1H, s). ^{13}C NMR (126 MHz, DMSO-d_6) 163.7 (C), 162.0 (C), 158.6 (CH), 158.4 (C), 138.7 (C), 121.7 (CH), 117.0 (C), 108.3 (CH), 104.9 (CH), 24.3 (CH), 6.9 (CH_2). HRMS: Found 244.1192, calculated for $C_{12}H_{14}N_5O$ $(M+H)^+$: 244.1193.

*3-Amino-5-(pyridin-3-yl)pyridin-2(1H)-one (**11**)*

Prepared using general procedure B to give a grey solid (24 mg, 65%): 6-Methoxy-3,3'-bipyridin-5-amine (40 mg, 0.199 mmol, 1 equivalent), trimethylsilyl chloride (0.994 mL, 0.994 mmol, 5 equivalents) and sodium iodide (149 mg, 0.994 mmol, 5 equivalents) in acetonitrile (1 mL). R_f = 0.2 (5% MeoOH in EtOAc). mp: 223 °C. ^1H NMR (500 MHz, DMSO-d_6) 5.22 (2H, s), 6.81 (1H, d, J = 2.4 Hz), 7.06 (1H, d, J = 2.4 Hz), 7.40 (1H, ddd, J = 0.8 Hz, 4.8 Hz, 8.0 Hz), 7.87 (1H, ddd, J = 1.6 Hz, 2.4 Hz, 8.0 Hz), 8.46 (1H, dd, J = 1.6 Hz, 4.7 Hz), 8.71 (1H, dd, J = 0.8 Hz, 2.4 Hz). ^{13}C NMR (126 MHz, DMSO-d_6) 157.7 (C), 147.9 (CH), 146.9 (CH), 139.5 (C), 133.6 (C), 133.1 (CH), 124.2 (C), 118.7 (CH), 116.5 (C), 109.5 (CH). HRMS: Found 188.0816, calculated for $C_{10}H_{10}N_3O$ $(M+H)^+$: 188.0824.

5-(4-((Dimethylamino)methyl)phenyl)-2-methoxypyridin-3-amine

2-Methoxy-3-amino-5-(4,4,5,5-tetramethyl-1,3,2-dioxaborolan-2-yl)pyridine (200 mg, 0.80 mmol, 1 equivalent) was dissolved in toluene (8 mL) and ethanol (2 mL) and then 1-(4-bromophenyl)-N,N-dimethylmethylamine (205 mg, 0.96 mmol, 1.2 equiv-alents) was added followed by tetrakis(triphenylphosphine)palladium(0) (46.2 mg, 0.04 mmol, 0.05 equivalents). Sodium carbonate (585 mg, 5.52 mmol, 6.9 equivalents) and water (3.5 mL) were then added and the reaction mixture heated to reflux for 2 hours under argon. After cooling the solution was diluted with water (15 mL) and extracted with EtOAc (3x50 mL) and the organic layers collected and washed with brine. The organic layers were then dried over magnesium sulfate and concentrated. The residue was purified using an SCX column (eluted with 2 M ammonia in methanol) to give a grey solid (147 mg, 71.4%). R_f = 0.24 (5%

MeOH in EtOAc). ^{1}H NMR (500 MHz, CD_3OD) 2.86 (6H, s), 4.02 (3H, s), 4.31 (2H, s), 7.27 (1H, d, J = 2.2 Hz), 7.56 (2H, d, J = 8.3 Hz), 7.69 (2H, d, J = 8.3 Hz), 7.72 (1H, d, J = 2.2 Hz).^{13}C NMR (126 MHz, CD_3OD) 152.8 (C), 140.0, 132.2 (C), 131.4 (CH), 131.0 (CH), 129.8 (C), 127.0 (CH), 118.1 (CH), 113.1 (C), 60.7 (CH_2), 52.6 (CH_3), 41.7 (CH_3). HRMS: Found 258.1598, calculated for $C_{15}H_{20}N_3O$ $(M+H)^+$: 258.1601.

*3-Amino-5-(4((dimethylamino)methyl)phenyl)aminopyridin-2-one (**12**)*

Prepared using general procedure B to give a grey solid (18 mg, 64%): 5-(4-((dimethylamino)methyl)phenyl)-2-methoxypyridin-3-amine (30 mg, 0.12 mmol, 1 equivalent), trimethylsilyl chloride (0.075 mL, 0.58 mmol, 5 equivalents) and sodium iodide (87 mg, 0.58 mmol, 5 equivalents) in acetonitrile (1 mL) and THF (1 mL). R_f = 0.22 (10% MeOH in $CHCl_3$). mp: 251 °C. ^{1}H NMR (500 MHz, DMSO-d_6) 2.15 (6H, s), 3.37 (2H, s), 5.16 (2H, s), 6.81 (1H, d, J = 2.4 Hz), 6.93 (1H, d, J = 2.4 Hz), 7.29 (2H, d, J = 8.3 Hz), 7.41 (2H, d, J = 8.2 Hz), 11.53 (1H, s).^{13}C NMR (126 MHz, DMSO-d_6) 157.5 (C), 139.2 (C), 137.7 (C), 136.6 (C), 129.7 (CH), 125.4 (CH), 119.4 (C), 117.7 (CH), 110.2 (CH), 63.5 (CH_2), 45.47 (CH_3). HRMS: Found 244.1443, calculated for $C_{14}H_{18}N_3O$ $(M+H)^+$: 244.1444.

*6-Methyl-2-oxo-5-(pyridin-4-yl)-1,2-dihydropyridine-3-carboxamide (**14**)*

Concentrated sulfuric acid (350 μL, 6.3 mmol, 14 equivalents) was added to 6-methyl-2-oxo-5-(pyridin-4-yl)-1,2-dihydropyridine-3-carbonitrile (95 mg, 0.450 mmol, 1 equivalent), and the solution was heated to 120 °C for 15 minutes. The solution was then cooled to 0 °C and diluted with water, whereupon concentrated ammonium hydroxide was added to bring the pH to 10. The resulting precipitate was filtered and dried to give the desired product as a white solid (74 mg, 72%). R_f = 0.53 (DCM:EtOAc 1:1). mp: 286 °C. ^{1}H NMR (500 MHz, DMSO-d_6) 2.34 (3H, s), 7.42 (2H, dd, J = 1.5 Hz, 4.5 Hz), 7.60 (1H, d, J = 3.8 Hz), 8.21 (1H, s), 8.62 (2H, dd, J = 1.5 Hz, 4.5 Hz), 8.97 (1H, d, J = 3.8 Hz), 11.09 (1H, s). ^{13}C NMR (126 MHz, DMSO-d_6) 164.9 (C), 162.6 (C), 150.3 (CH), 149.3 (C), 145.3 (CH), 145.1 (C), 124.4 (CH), 118.6 (C), 116.6 (C), 18.2 (CH_3). HRMS: Found 230.0923, calculated for $C_{12}H_{12}N_3O_2$ $(M+H)^+$: 230.0924.

*3-Amino-5-(pyridin-4-yl)-6-methylpyridin-2-one (**15**)*

6-Methyl-2-oxo-5-(pyridin-4-yl)-1,2-dihydropyridine-3-carboxamide (60 mg, 0.262 mmol, 1 equivalent) was dissolved in 1 M NaOH (1.5 mL) with heating and then cooled to 0 °C. Bromine (16 μL, 0.314 mmol, 1.2 equivalents) was then added dropwise and the reaction mixture heated to 120 °C for 3 hours then cooled. The solution was then adjusted to pH = 7 with HCl (1 M) and cooled to 0 °C for 2 hours. The precipitate was then filtered and washed with water to give the desired product as an off-white solid (12 mg, 23%). R_f = 0.62 (5% MeOH in EtOAc). mp: 262 °C. ^{1}H NMR (500 MHz, DMSO-d_6) 2.12 (3H, s), 4.97 (2H, s), 6.47 (1H, s), 7.30 (2H, d, J = 5.2 Hz), 8.55 (2H, brs), 11.56 (1H, brs).^{13}C NMR (126 MHz, DMSO-d_6) 158.3 (C), 150.0 (N**C**HCH), 147.2 (C), 137.1 (C), 127.8 (C), 124.3 (CH), 115.4 (C), 113.1 (CH), 16.8 (CH_3). HRMS: Found 202.0985, calculated for $C_{11}H_{12}N_3O$ $(M+H)^+$: 202.0975.

*3,5-Difluoro-N-(2-oxo-5-(pyridin-4-yl)-1,2-dihydropyridin-3-yl)benzamide (**20**)*

Prepared using general procedure C to give a yellow solid (48 mg, 54%): 3,5-difluorobenzoylchloride (0.031 mL, 0.27 mmol, 1 equivalent), 3-amino-5-(pyridin-4-yl)pyridin-2-one (50 mg, 0.27 mmol, 1 equivalent). R_f = 0.51 (DCM:EtOAc 1:1). mp: 293 °C. ^{1}H NMR (500 MHz, DMSO-d_6) 7.56 (1H, tt, J = 2.1 Hz, 9.0 Hz), 7.65 - 7.72 (2H, m), 8.21 (2H, d, J = 6.7 Hz), 8.27 (1H, s), 8.76 (1H, d, J = 2.6 Hz), 8.83 (2H, d, J = 6.6 Hz), 9.77 (1H, s), 13.00 (1H, s). ^{13}C NMR (126 MHz, DMSO-d_6) 163.8 (C), 162.7 (dd, J = 247.8 Hz, 12.8 Hz, CF), 158.1 (C), 152.0 (C), 142.9 (CH), 137.8 (t, J = 8.6 Hz, C), 133.4 (CH), 129.2 (C), 124.6 (CH), 121.9 (CH), 112.9 (C), 111.6 (dd, J = 20.4 Hz, 6.5 Hz, CH), 108.0 (t, J = 25.9 Hz, C). HRMS: Found 328.0893, calculated for $C_{17}H_{12}N_3O_2F_2$ $(M+H)^+$: 328.0892.

*N-(2-Oxo-5-(pyridin-4-yl)-1,2-dihydropyridin-3-yl)benzenesulfonamide (**21**)*

Prepared using general procedure C to give a yellow solid (18 mg, 21%): benzenesulfonylchloride (0.034 mL, 0.27 mmol, 1 equivalent), 3-amino-5-(pyridin-4-yl)pyridin-2-one (50 mg, 0.27 mmol, 1 equivalent). R_f = 0.31 (5% MeOH in EtOAc). mp: 289 °C. ^{1}H NMR (500 MHz, DMSO-d_6) 7.52 (2H, dd, J = 1.6 Hz, 4.6 Hz), 7.56 (2H, t, J = 7.6 Hz), 7.61 - 7.66 (1H, m), 7.74 (1H, d, J = 2.5 Hz), 7.76 (1H, d, J = 2.5 Hz), 7.87 - 7.93 (2H, m), 8.57 (2H, dd, J = 1.6 Hz, 4.6 Hz), 9.76 (1H, s), 12.34 (1H, s).^{13}C NMR (126 MHz, DMSO-d_6) 157.8 (C), 150.6, 150.0 (CH), 143.8 (C), 140.3 (C), 133.6 (CH), 129.7 (CH), 128.6 (C), 127.3 (CH), 124.5 (CH), 124.4 (CH), 120.0 (CH). HRMS: Found: 328.0750, calculated for $C_{16}H_{14}N_3O_3S$ $(M+H)^+$: 328.0750.

4-Bromo-N-(2-oxo-5-(pyridin-4-yl)-1,2-dihydropyridin-3-yl)benzamide

Prepared using general procedure C to give a white solid (255 mg, 52%): 4-bromobenzoyl chloride (293 mg, 1.34 mmol, 1 equivalent), 3-amino-5-(pyridin-4-yl)pyridin-2-one (250 mg, 1.34 mmol, 1 equivalent). R_f = 0.58 (DCM:EtOAc 1:1). mp: 294 °C. ^{1}H NMR (500 MHz, DMSO-d_6) 7.78 (2H, d, J = 8.6 Hz), 7.91 (2H, d, J = 8.6 Hz), 8.13 (2H, d, J = 6.8 Hz), 8.19 (1H, s), 8.79 (2H, d, J = 6.8 Hz), 8.82 (1H, d, J = 2.6 Hz), 9.59 (1H, s), 12.94 (1H, d, J = 2.7 Hz).^{13}C NMR (126 MHz, DMSO-d_6) 165.1 (C), 158.1 (C), 151.3 (C), 143.8 (CH), 133.3 (C), 132.3 (CH), 132.0 (CH), 130.0 (CH), 129.5 (C), 126.5 (C), 123.1 (CH), 121.7 (CH), 113.4 (C).

4-(4-Methylpiperazin-1-yl)-N-(2-oxo-5-(pyridin-4-yl)-1,2-dihydropyridin-3-yl)benzamide (***22***)

General Procedure D: 4-Bromo-*N*-(2-oxo-5-(pyridin-4-yl)-1,2-dihydropyridin-3-yl) benzamide (100 mg, 0.27 mmol, 1 equivalent) and 1-methylpiperazine (3 mL, 27 mmol, 100 equivalents) were stirred in *N*-methyl-2-pyrrolidinone (1.5 mL) at 200 °C in a microwave reactor for 6 hours. The excess 1-methylpiperazine was then removed under reduced pressure and the product filtered and washed with methanol to give a white solid (38 mg, 36%). R_f = 0.70 (DCM:EtOAc 1:1). mp: 280 °C. ^{1}H NMR (500 MHz, DMSO-d_6) 2.23 (3H, s), 2.42 - 2.47 (4H, m), 3.26 - 3.32 (4H, m), 7.05 (2H, d, J = 9.0 Hz), 7.60 (2H, dd, J = 1.6 Hz, 4.6 Hz), 7.77 (1H, d, J = 2.5 Hz), 7.80 (2H, d, J = 9.0 Hz), 8.58 (2H, dd, J = 1.6 Hz, 4.5 Hz), 8.77 (1H, d, J = 2.5 Hz), 9.15 (1H, s), 12.54 (1H, s). ^{13}C NMR (126 MHz, DMSO-d_6) 165.2 (C), 157.9 (C), 153.9 (C), 150.7 (CH), 144.3 (C), 129.9 (C), 129.1 (CH), 127.1 (CH) , 122.6 (C), 121.3 (CH), 120.3 (CH), 115.8 (C), 114.2 (CH), 54.7 (CH_2), 47.2 (CH_2), 46.2 (CH_3). HRMS: Found: 390.1925, calculated for $C_{22}H_{24}N_5O_2$ $(M+H)^+$: 390.1917.

N-(2-Oxo-5-(pyridin-4-yl)-1,2-dihydropyridin-3-yl)-4-piperidin-1-yl)benzamide (**23**)

Prepared using general procedure D to give a white solid (26 mg, 26%): 4-bromo-*N*-(2-oxo-5-(pyridin-4-yl)-1,2-dihydropyridin-3-yl)benzamide (100 mg, 0.27 mmol, 1 equivalent), piperidine (2.67 mL, 27 mmol, 100 equivalents). R_f = 0.71 (DCM: EtOAc 1:1). mp: 284 °C. ^{1}H NMR (500 MHz, DMSO-d_6) 1.59 (6H, brs), 3.34 (4H, brs), 7.01 (2H, d, J = 8.9 Hz), 7.60 (2H, d, J = 6.1 Hz), 7.76 (1H, d, J = 2.5 Hz), 7.77 (2H, d, J = 9.0 Hz), 8.58 (2H, d, J = 6.0 Hz), 8.77 (1H, d, J = 2.5 Hz), 9.12 (1H, s), 12.54 (1H, s).^{13}C NMR (126 MHz, DMSO-d_6) 165.1 (C), 157.8 (C), 153.9 (C), 150.7 (CH), 144.2 (C), 130.0 (C), 129.1 (CH), 126.9 (CH), 121.7 (C), 120.9 (CH), 120.2

(CH), 115.8 (C), 114.1 (CH), 48.4 (CH_2), 25.3 (CH_2), 24.4 (CH_2). HRMS: Found: 375.1799, calculated for $C_{22}H_{23}N_4O_2$ $(M+H)^+$: 375.1816.

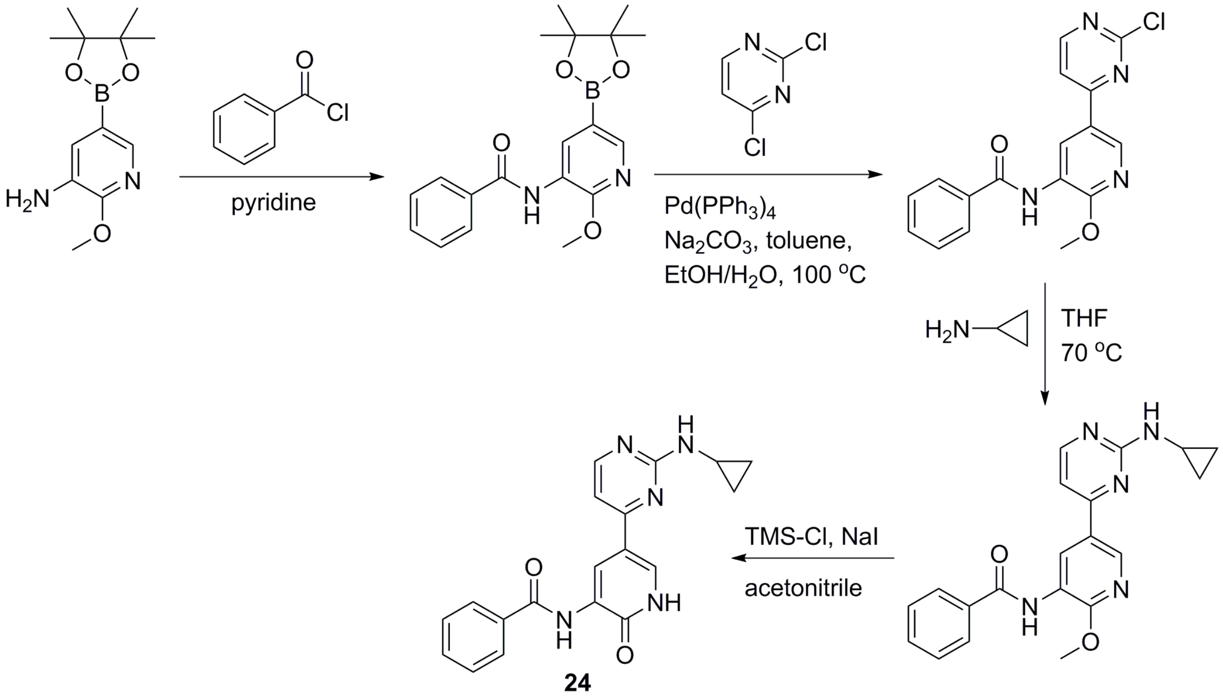

24

Figure 3: Synthesis of *N*-(5-(2-(cyclopropylamino)pyrimidin-4-yl)-2-oxo-1,2-dihydropyridin-3-yl)benzamide (**24**).

N-(2-Methoxy-5-(4,4,5,5-tetramethyl-1,3,2-dioxaborolan-2-yl)pyridin-3-yl)benzamide

Benzoyl chloride (0.058 mL, 0.5 mmol, 1 equivalent) was added to a solution of 2-methoxy-3-amino-5-(4,4,5,5-tetramethyl-1,3,2-dioxaborolan-2-yl)pyridine (125 mg, 0.5 mmol, 1 equivalent) in pyridine (3.5 mL) and stirred for 16 hours. The solvent was then removed under reduced pressure and the residue was taken up methanol and filtered to give the product as a white solid (87 mg, 49%). R_f = 0.54 (DCM:EtOAc 1:1). mp: 251 °C. ^{1}H NMR (500 MHz, $CDCl_3$) 1.35 (12H, s), 4.10 (3H, s), 7.49 - 7.62 (3H, m), 7.91 (2H, d, J = 8.0 Hz), 8.28 (1H, s), 8.37 (1H, s), 9.06 (1H, s).^{13}C (126 MHz, $CDCl_3$) 165.5 (C), 155.2 (C), 147.5 (CH), 134.7 (C), 132.3 (CH), 132.0 (CH), 128.9 (CH), 127.0 (CH), 122.4 (C), 83.9 (C), 54.2 (CH_3), 24.9 (CH_3). HRMS: Found 377.1640, calculated for $C_{19}H_{23}N_2O_4BNa$ $(M+Na)^+$: 377.1647.

N-(5-(2-Chloropyrimidin-4-yl)-2-methoxypyridin-3-yl)benzamide

N-(2-Methoxy-5-(4,4,5,5-tetramethyl-1,3,2-dioxaborolan-2-yl)pyridin-3-yl)benzamide (175 mg, 0.494 mmol, 1 equivalent), 2,4-dichloropyrimidine (88 mg, 0.593 mmol, 1.2 equivalents) and tetrakis(triphenylphosphine)palladium(0) (28.5 mg, 0.025 mmol,

0.05 equivalents) were added to toluene (8 mL) and ethanol (2 mL) followed by addition of sodium carbonate (361 mg, 3.41 mmol, 6.9 equivalents) and water (3 mL). The reaction mixture was then heated to reflux for 2 hours and the solvent removed under reduced pressure and the residue purified by column chromatography (20% EtOAc in DCM) to give a pale yellow solid (80 mg, 48%). R_f = 0.71 (20% EtOAc in DCM). mp: >300 °C. ^{1}H NMR (500 MHz, $CDCl_3$) 4.18 (3H, s), 7.53 - 7.60 (2H, m), 7.60 - 7.65 (1H, m), 7.68 (1H, d, J = 5.3 Hz), 7.91 - 7.96 (2H, m), 8.48 (1H, s), 8.65 (1H, d, J = 5.3 Hz), 8.75 (1H, d, J = 2.3 Hz), 9.44 (1H, d, J = 2.3 Hz).^{13}C NMR (126 MHz, $CDCl_3$) 168.6 (C), 159.9 (CH), 140.7 (CH), 132.6 (CH), 129.0 (CH), 127.2 (CH), 125.3 (C), 124.4 (CH), 123.2 (C), 114.7 (CH), 54.7 (CH_3). HRMS: Found 341.0805, calculated for $C_{17}H_{14}N_4O_2Cl$ $(M+H)^+$: 341.0800.

N-(5-(2-(Cyclopropylamino)pyrimidin-4-yl)-2-methoxypyridin-3-yl)benzamide

Prepared by general procedure D to give a yellow solid (19 mg, 51%): *N*-(5-(2-chloropyrimidin-4-yl)-2-methoxypyridin-3-yl)benzamide (35 mg, 0.10 mmol, 1 equivalent), cyclopropylamine (0.142 mL, 2.05 mmol, 20 equivalents) and THF (1 mL). The reaction mixture was stirred in a sealed vial at 70 ºC for 24 hours. R_f = 0.52 (DCM:EtOAc 1:1). ^{1}H NMR (500 MHz, $CDCl_3$) 0.59 - 0.66 (2H, m), 0.84 - 0.94 (2H, m), 2.72 - 3.02 (1H, m), 4.15 (3H, s), 5.43 (1H, s), 7.06 (1H, d, J = 5.2 Hz), 7.55 (2H, t, J = 7.4 Hz), 7.61 (1H, t, J = 7.4 Hz), 7.91 - 7.97 (2H, m), 8.41 (1H, d, J = 5.2 Hz), 8.45 (1H, s), 8.65 (1H, s), 9.45 (1H, d, J = 2.1 Hz). HRMS: Found 362.1607, calculated for $C_{20}H_{20}N_5O_2$ $(M+H)^+$: 362.4051.

*N-(5-(2-(Cyclopropylamino)pyrimidin-4-yl)-2-oxo-1,2-dihydropyridin-3-yl)benzamide (**24**)*

Prepared using general procedure B to give a yellow solid (12 mg , 65%): *N*-(5-(2-(cyclopropylamino)pyrimidin-4-yl)-2-methoxypyridin-3-yl)benzamide (19 mg, 0.053 mmol, 1 equivalent), sodium iodide (23.64 mg, 0.158 mmol, 3 equivalents), trimethylsilyl chloride (0.02 mL, 0.158 mmol, 3 equivalents) and acetonitrile 0.5 mL). R_f = 0.52 (5% MeOH in EtOAc). mp: 292 °C. ^{1}H NMR (500 MHz, DMSO-d_6) 0.38 - 0.59 (2H, m), 0.59 - 0.74 (2H, m), 2.63 - 2.87 (1H, m), 6.99 (1H, d, J = 5.2 Hz), 7.29 (1H, d, J = 3.2 Hz), 7.56 (2H, t, J = 7.3 Hz), 7.62 (1H, t, J = 7.3 Hz), 7.93 (2H, m), 8.14 (1H, brs), 8.25 (1H, d, J = 5.2), 8.98 (1H, brs), 9.41 (1H, brs).^{13}C NMR (126 MHz, DMSO-d_6) 165.0 (C), 163.7 (C), 158.6 (CH), 134.6 (C), 132.3 (CH), 129.1 (CH), 127.5 (CH), 121.3 (CH), 115.8 (C), 104.5 (CH), 24.5 (CH), 7.1 (CH_2). HRMS: Found 348.1453, calculated for $C_{19}H_{18}N_5O_2$ $(M+H)^+$: 348.1455.

Mobility Shift Assay Protocol

Screening of 26 kinases using a mobility shift assay

A mobility shift assay to determine kinase inhibition was carried out using a modified version of the ProfilerPro assay kit 1 (Perkin Elmer, Coventry, UK) including MPS1 and Aurora B. Each kit contained an enzyme plate, a substrate plate, reconstitution buffer, 1 M DTT, protease inhibitor cocktail and termination buffer. Test compounds were added as a solution in DMSO. The 3-aminopyridin-2-one compounds **1-3, 5-8** and **15** were tested at 100 μM final concentration. The 3-benzamido-pyridin-2-one compounds **16, 22,** and **24** were tested at 30 μM final concentration. Compound **17** was tested at 100 μM final concentration. The peptide phosphorylation assay was carried out as per the manufacturer's instructions. The electrophoretic separation and analysis of the phosphorylated and non-phosphorylated peptides was carried out on a 4-sipper chip using a LabChip EZReader II (Perkin Elmer). Results were expressed as % inhibition of peptide phosphorylation relative to control wells for 0 % (no test compound) and 100 % (no ATP) inhibition. The percentage inhibition was calculated using the following equation:

$$\% \text{ Inhibition} = [1 - (\% \text{ Conversion}_{\text{Sample}} / \% \text{ Conversion}_{0\% \text{ Inhibition Control}})] \times 100$$

Kinase	[ATP] (μM)	[Enzyme] (nM)	[Peptide Substrate] (μM)	$MgCl_2$ (mM)
MPS1	10.0	6.0	5.0	10
Aurora A	20.0	5.0	1.5	5
Aurora B	15.0	1.0	1.5	5
SRC	38.0	0.219	1.5	10
RSK1	23.3	5.582	1.5	10
MAPKAPK5	5.0	3.143	1.5	10
PKD2	32.1	0.445	1.5	10
PKCζ	3.8	3.34	1.5	10
PKA	1.7	0.437	1.5	10
p38α	396.5	3.733	1.5	10
MSK1	21.2	2.507	1.5	10
MET	79.5	2.819	1.5	10
MAPKAPK2	4.6	0.081	1.5	10
LYN	17.0	0.976	1.5	10
LCK	28.5	0.187	1.5	10
INSR	871.8	21.367	1.5	10
GSK3β	7.3	3.211	1.5	10
FYN	36.0	0.197	1.5	10
Erk2	62.1	1.298	1.5	10
Erk1	33.4	1.051	1.5	10
CK1d	16.3	2.416	1.5	10
CHK2	57.8	8.389	1.5	10
CHK1	33.0	2.805	1.5	10
AKT2	186.1	0.293	1.5	10
AKT1	48.0	0.174	1.5	10
ABL	14.0	0.401	1.5	10

Table 1: Assay Conditions for Mobility Shift Assay

Kinase	% Inhibition							
	1	2	3	5	6	7	8	15
MPS1	0	41	50	41	69	71	87	20
AurA	58	100	0	46	62	98	87	49
AurB	20	96	43	35	61	94	79	77
SRC	31	86	12	8	24	89	46	21
RSK1	19	89	27	23	10	91	89	17
PRAK	19	99	0	1	15	88	24	24
PKD2	10	67	0	26	36	70	81	8
PKCζ	27	83	50	5	17	37	0	13
PKA	0	66	68	52	48	81	8	0
p38α	9	39	0	0	0	25	12	8
MSK1	26	95	27	61	22	94	27	14
MET	33	81	0	18	19	76	26	14
MAPKAPK2	25	97	1	19	32	79	28	33
LYN	15	49	18	15	27	63	42	10
LCK	4	11	6	7	40	55	38	4
INSR	2	25	1	6	13	80	27	23
GSK3β	17	68	9	26	20	89	87	39
FYN	11	34	10	9	40	66	51	6
Erk2	3	68	0	12	15	53	45	11
Erk1	9	62	0	20	22	52	46	11
CK1d	45	72	40	38	37	79	63	33
CHK2	49	99	0	36	57	98	61	42
CHK1	37	77	68	1	26	79	26	4
AKT2	77	97	0	4	9	93	18	17
AKT1	42	97	12	22	15	89	18	10
ABL	5	58	11	26	66	67	43	5
**S(30%)**	0.31	0.92	0.23	0.27	0.42	0.96	0.58	0.23
**S(50%)**	0.08	0.77	0.08	0.08	0.19	0.92	0.35	0.04
**S(80%)**	0.00	0.46	0.00	0.00	0.00	0.42	0.19	0.00

Table 2: % Inhibition for 3-aminopyridin-2-one fragment library at 100 μM measured using a microfluidics-based assay

	% Inhibition			
**Kinase**	**16**	**17 ***	**22**	**24**
MPS1	62	42	88	76
AurA	59	89	92	99
AurB	24	27	71	91
SRC	60	59	98	99
RSK1	19	38	41	98
PRAK	1	10	10	71
PKD2	30	22	46	69
PKCζ	0	0	24	0
PKA	0	6	0	75
p38α	4	3	6	36
MSK1	1	15	8	83
MET	9	23	24	65
MAPKAPK2	29	9	12	96
LYN	55	56	93	86
LCK	45	50	97	75
INSR	13	21	30	27
GSK3β	92	93	94	99
FYN	59	59	95	93
Erk2	5	14	1	37
Erk1	12	2	0	33
CK1d	79	78	31	78
CHK2	29	58	33	71
CHK1	24	21	84	0
AKT2	4	50	13	91
AKT1	12	24	13	73
ABL	55	75	96	88
**S(30%)**	0.35	0.46	0.54	0.88
**S(50%)**	0.31	0.31	0.38	0.77
**S(80%)**	0.04	0.08	0.35	0.38

Table 3: % Inhibition for 3-benzamido-pyridin-2-one based fragment library at 30 μM (*100 μM) measured using a microfluidics-based assay

Kinase	$S_{kinase}(50\%)$ 3-Aminopyridinone Fragment Library	$S_{kinase}(50\%)$ 3-Benzamidopyridinone Library
MPS1	0.38	0.75
AurA	0.63	1.00
AurB	0.63	0.50
SRC	0.25	1.00
RSK1	0.38	0.25
PRAK	0.25	0.25
PKD2	0.38	0.25
PKCζ	0.13	0.00
PKA	0.50	0.25
p38α	0.00	0.00
MSK1	0.38	0.25
MET	0.25	0.25
MAPKAPK2	0.25	0.25
LYN	0.13	1.00
LCK	0.13	0.50
INSR	0.13	0.00
GSK3β	0.38	1.00
FYN	0.25	1.00
Erk2	0.25	0.00
Erk1	0.25	0.00
CK1d	0.38	0.75
CHK2	0.50	0.50
CHK1	0.38	0.25
AKT2	0.38	0.25
AKT1	0.25	0.25
ABL	0.38	1.00

Table 4: $S_{kinase}(50\%)$ of 3-aminopyridin-2-one fragment library and 3-benzamidopyridin-2-one library

IC_{50} Determination

For all compounds, IC_{50} values for MPS1, Aurora A, and Aurora B were determined using MPS1 and Aurora A/B mobility shift assays as described previously.[1] IC_{50} values were calculated from a four-parameter logistic fit of percentage inhibition versus concentration (at 8 compound concentrations) using GraphPad Prism 5 (GraphPad Software, Inc., CA, USA). Mean IC_{50} values were determined from two independent measurements each carried out in duplicate. K_i values were estimated from the mean IC_{50} values using the Cheng-Prusoff equation.

Crystallisation
Structure Solution and Refinement

X-ray diffraction data were collected at the Diamond Light Source, Oxford, UK on beamline I04 or I24. Data were integrated and merged using MOSFLM[2, 3] and AIMLESS.[4] The structures were solved by molecular replacement using PHASER[3, 5] and 2ZMC as the molecular replacement model with ligands and water removed. The protein-ligand structures were manually rebuilt in COOT[6] and refined with BUSTER[7] in iterative cycles. Ligand restraints were generated with Grade[8] and mogul.[9] The quality of the structures was assessed with MOLPROBITY.[10]

Compound	23	22	2
**PDB Code**	4CVA	4CV9	4CV8
**Space Group**	*I222*	*I222*	*I222*
**Lattice Constants**			
a (Å)	70.46	70.38	70.81
b (Å)	107.16	108.72	110.31
c (Å)	112.75	113.56	114.24
**Data Collection**			
X-ray Source	Diamond	Diamond	Diamond
Beamline	I04	I04	I24
Resolution Range (Å)	40.72 - 2.50	40.94 - 2.50	41.23 - 3.00
Highest Resolution Shell (Å)	2.60 - 2.50	2.60 - 2.50	3.18 - 3.00
Unique Reflections	15117 (1679)	15436 (1726)	9187 (1454)
Completeness (%)	99.9 (100.0)	99.4 (99.9)	99.5 (99.7)
Multiplicity	4.4 (4.5)	4.8 (5.0)	4.4 (4.4)
Rmerge	0.09 (1.8)	0.06 (1.4)	0.14 (1.9)
CC1/2[11]	0.998 (0.34)	0.999 (0.482)	0.992 (0.354)
Mosaicity	0.18	0.19	0.14
Mean(I/σ(I))	10.7 (0.8)	13.8 (0.9)	9.2 (1.0)
**Refinement**			
R_{factor} (%)	18.97	18.54	18.03
R_{free} (%)	21.50	23.23	22.56
No. amino acids	254	254	255
No. waters	30	32	6
No. ligands	1	1	1
No. Ethylene glycol	2	6	4
No. DMSO	2	1	0
**r.m.s. deviation**			
Bond lengths (Å)	0.01	0.01	0.01
Bond angles (°)	1.08	1.12	1.15
**Ramachandran Plot**			
Favoured (%)	95.98	95.18	93.98
Forbidden (%)	0.00	0.00	0.00

Table 5: *Data collection and refinement statistics*

^{1}H-NMR spectra

Compound **4**

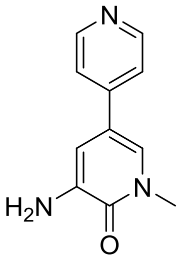

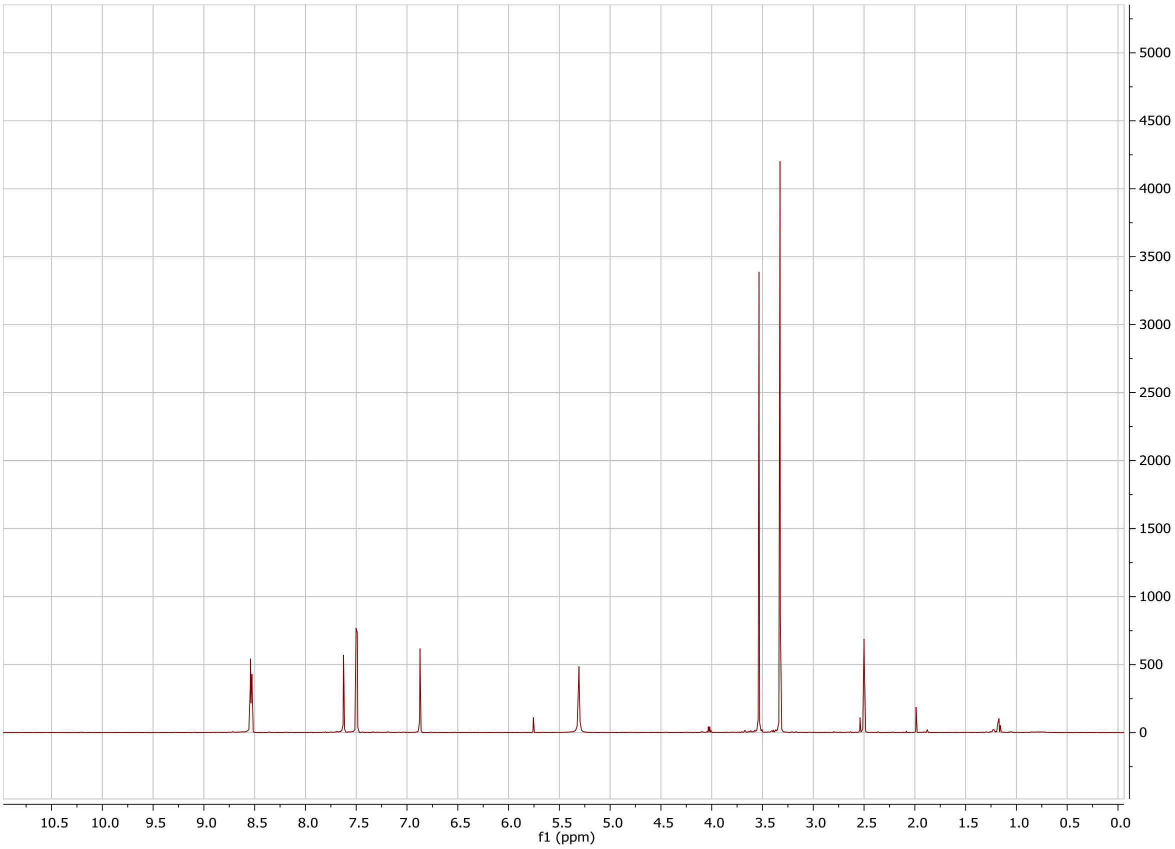

Compound **5**

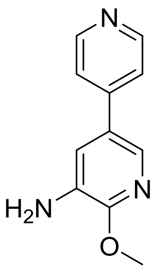
N
N
H2N
O

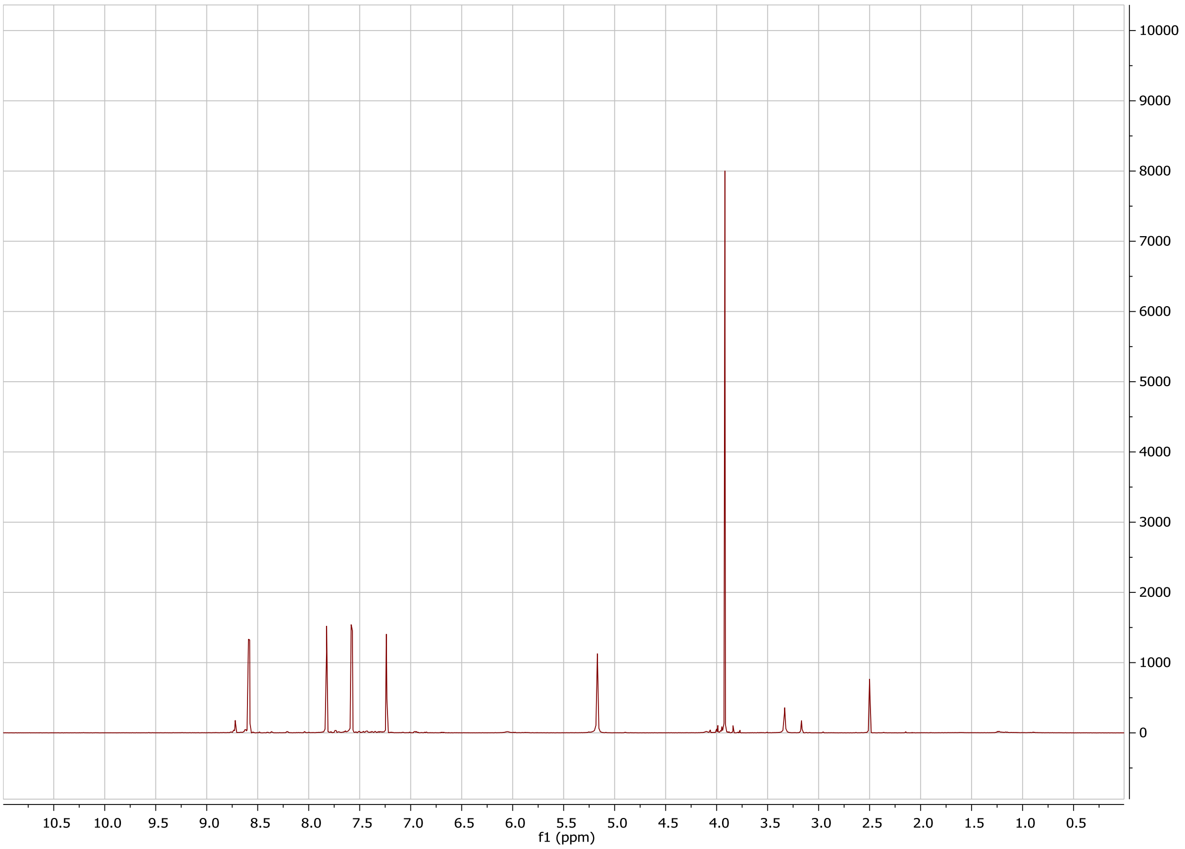
10000
9000
8000
7000
6000
5000
4000
3000
2000
1000
0
10.5
10.0
9.5
9.0
8.5
8.0
7.5
7.0
6.5
6.0
5.5
5.0
4.5
4.0
3.5
3.0
2.5
2.0
1.5
1.0
0.5
f1 (ppm)

Compound **7**

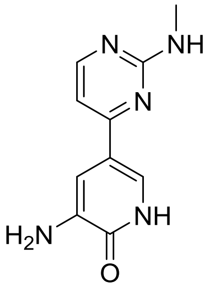

N
NH
N
NH
H2N
O

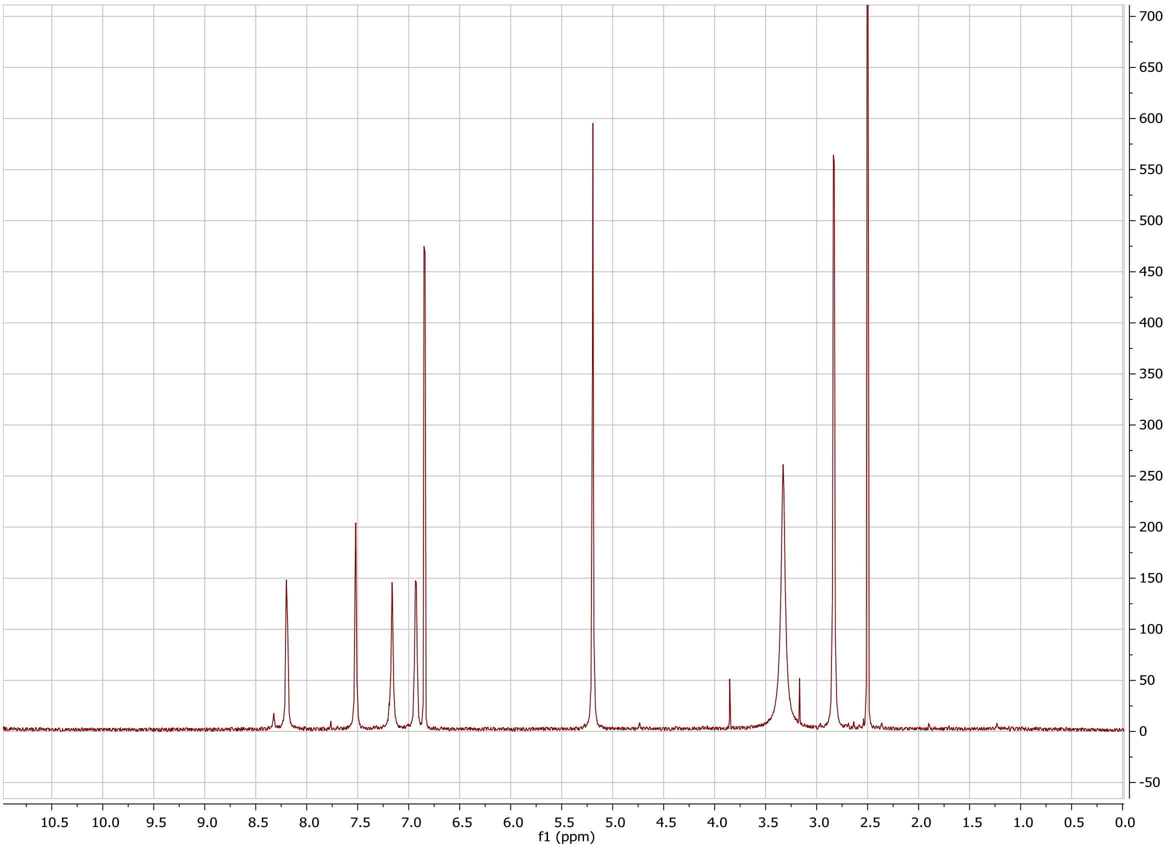

700
650
600
550
500
450
400
350
300
250
200
150
100
50
0
-50
10.5
10.0
9.5
9.0
8.5
8.0
7.5
7.0
6.5
6.0
5.5
5.0
4.5
4.0
3.5
3.0
2.5
2.0
1.5
1.0
0.5
0.0
f1 (ppm)

Compound **8**

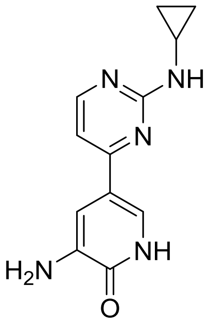

NH
N
N
NH
H2N
O

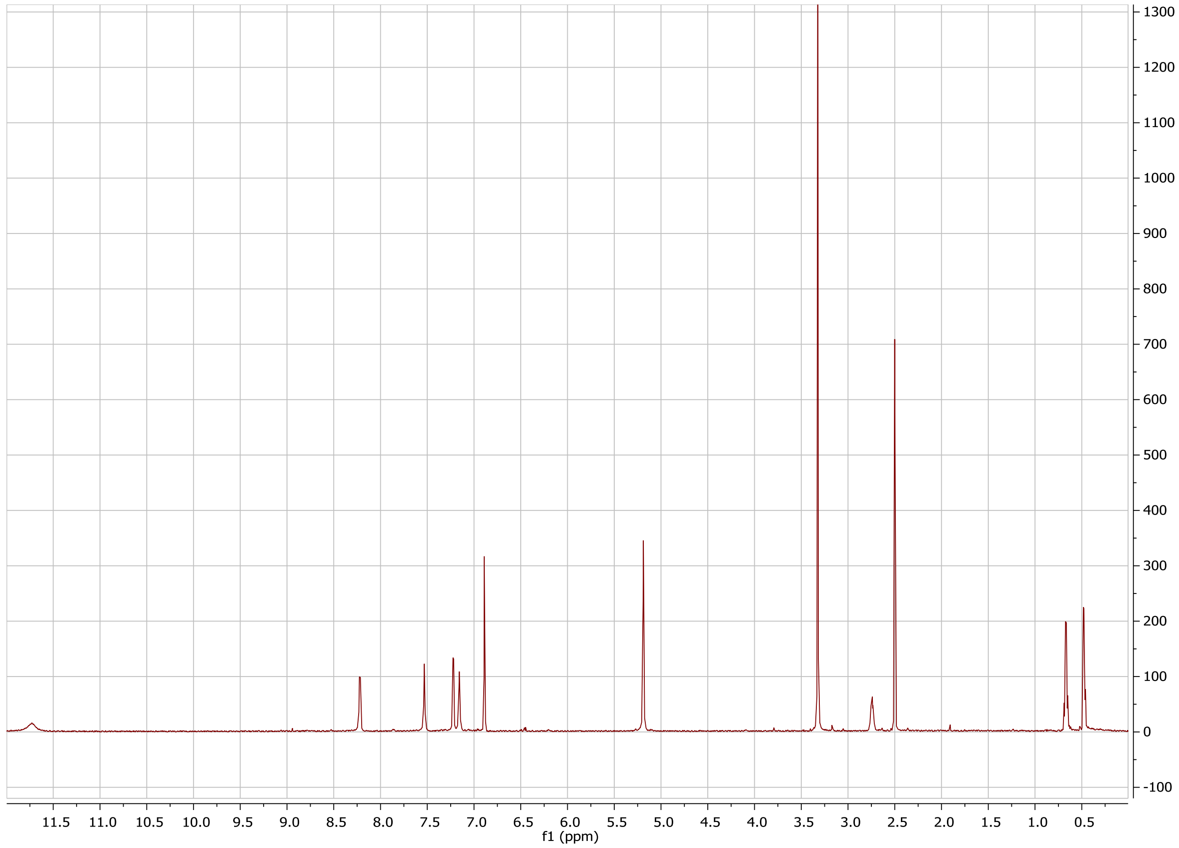

1300
1200
1100
1000
900
800
700
600
500
400
300
200
100
0
-100
11.5 11.0 10.5 10.0 9.5 9.0 8.5 8.0 7.5 7.0 6.5 6.0 5.5 5.0 4.5 4.0 3.5 3.0 2.5 2.0 1.5 1.0 0.5
f1 (ppm)

Compound **9**

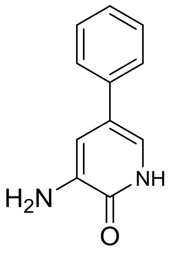

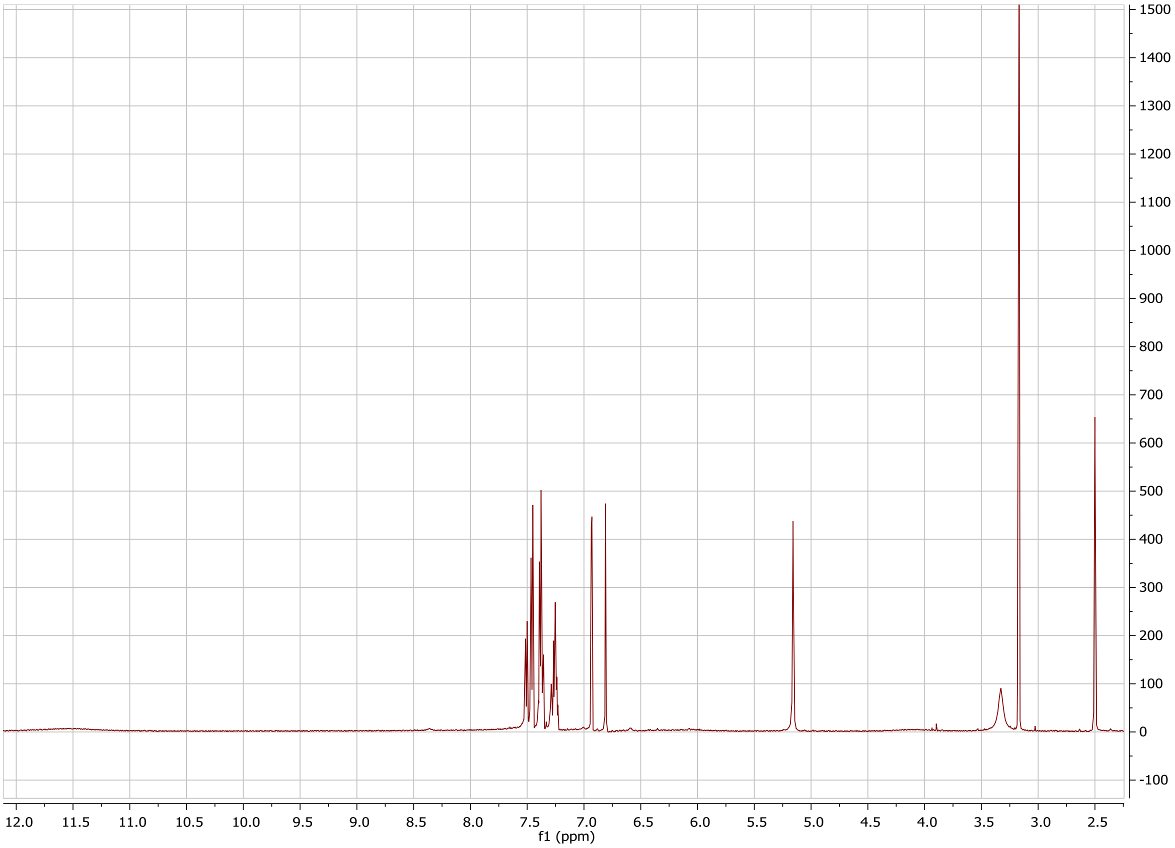

1500
1400
1300
1200
1100
1000
900
800
700
600
500
400
300
200
100
0
-100
12.0 11.5 11.0 10.5 10.0 9.5 9.0 8.5 8.0 7.5 7.0 6.5 6.0 5.5 5.0 4.5 4.0 3.5 3.0 2.5
f1 (ppm)

Compound **10**

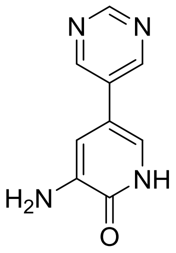
N
N
NH
H_2N
O

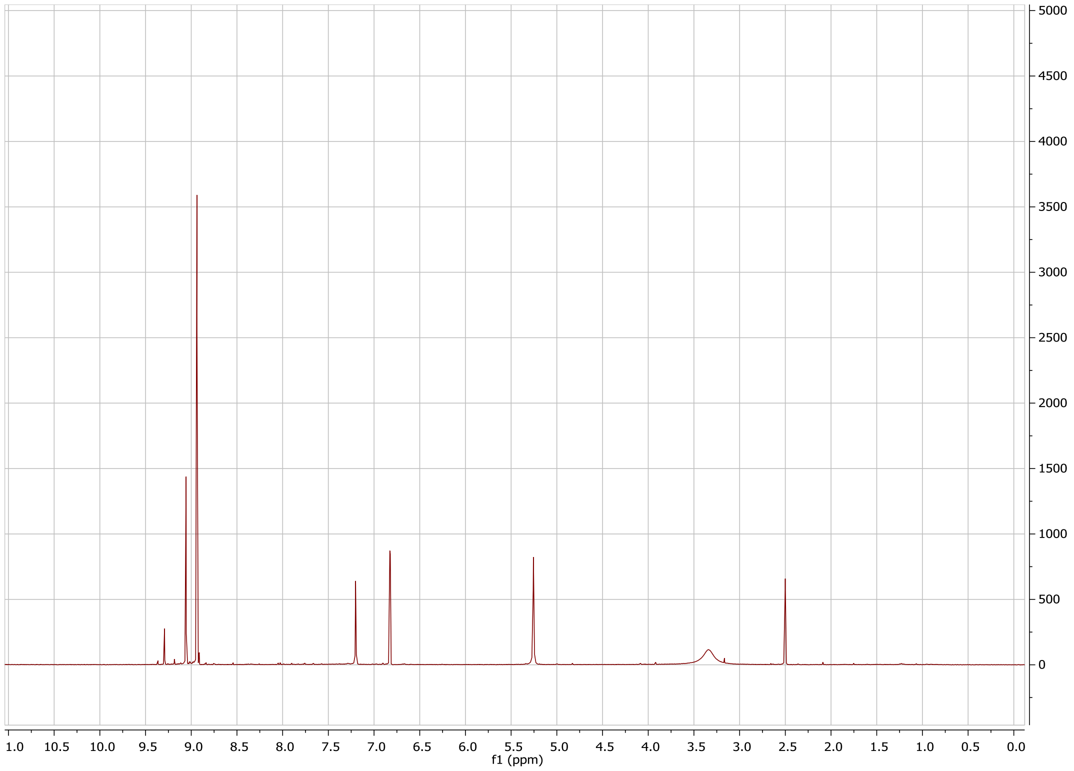
5000
4500
4000
3500
3000
2500
2000
1500
1000
500
0
1.0
10.5
10.0
9.5
9.0
8.5
8.0
7.5
7.0
6.5
6.0
5.5
5.0
4.5
4.0
3.5
3.0
2.5
2.0
1.5
1.0
0.5
0.0
f1 (ppm)

Compound **12**

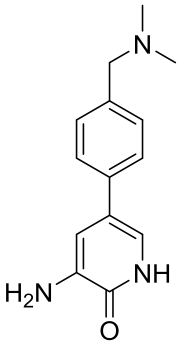

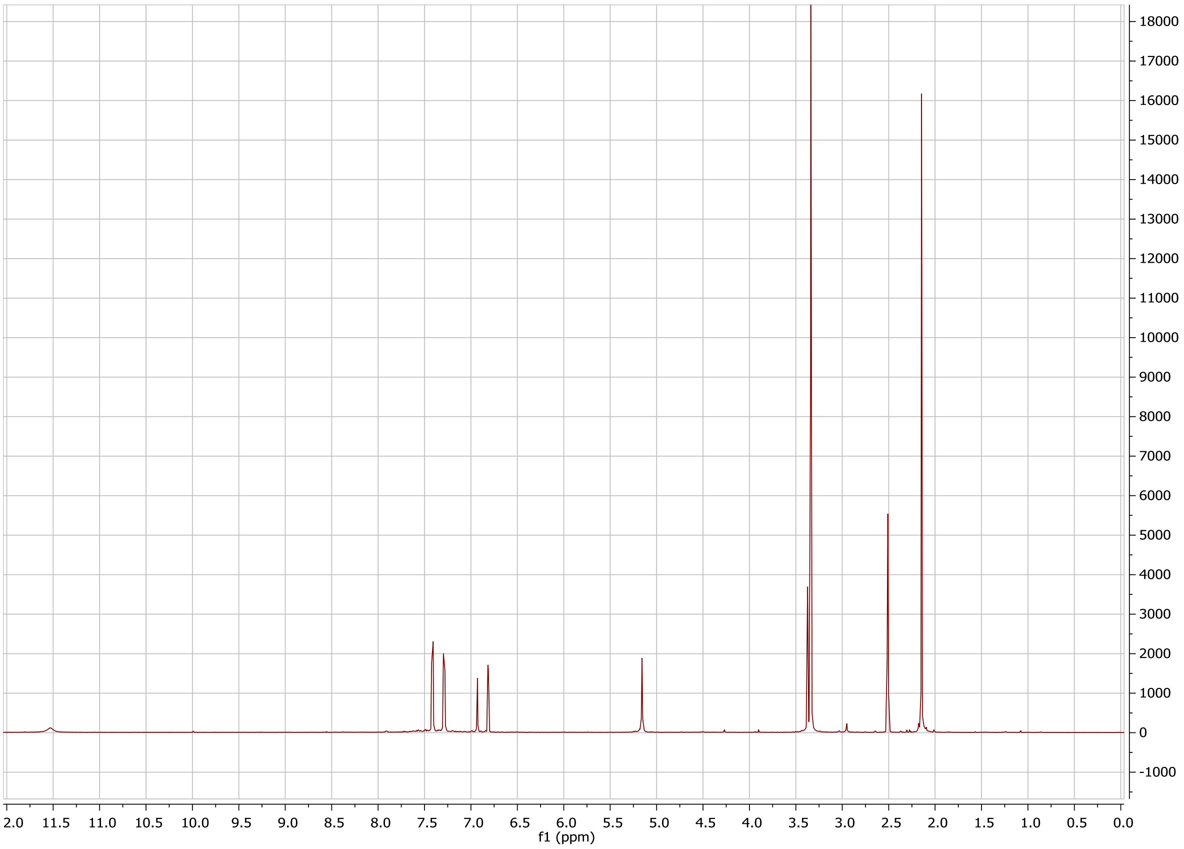

Compound **15**

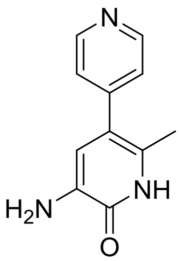
N
NH
H_2N
O

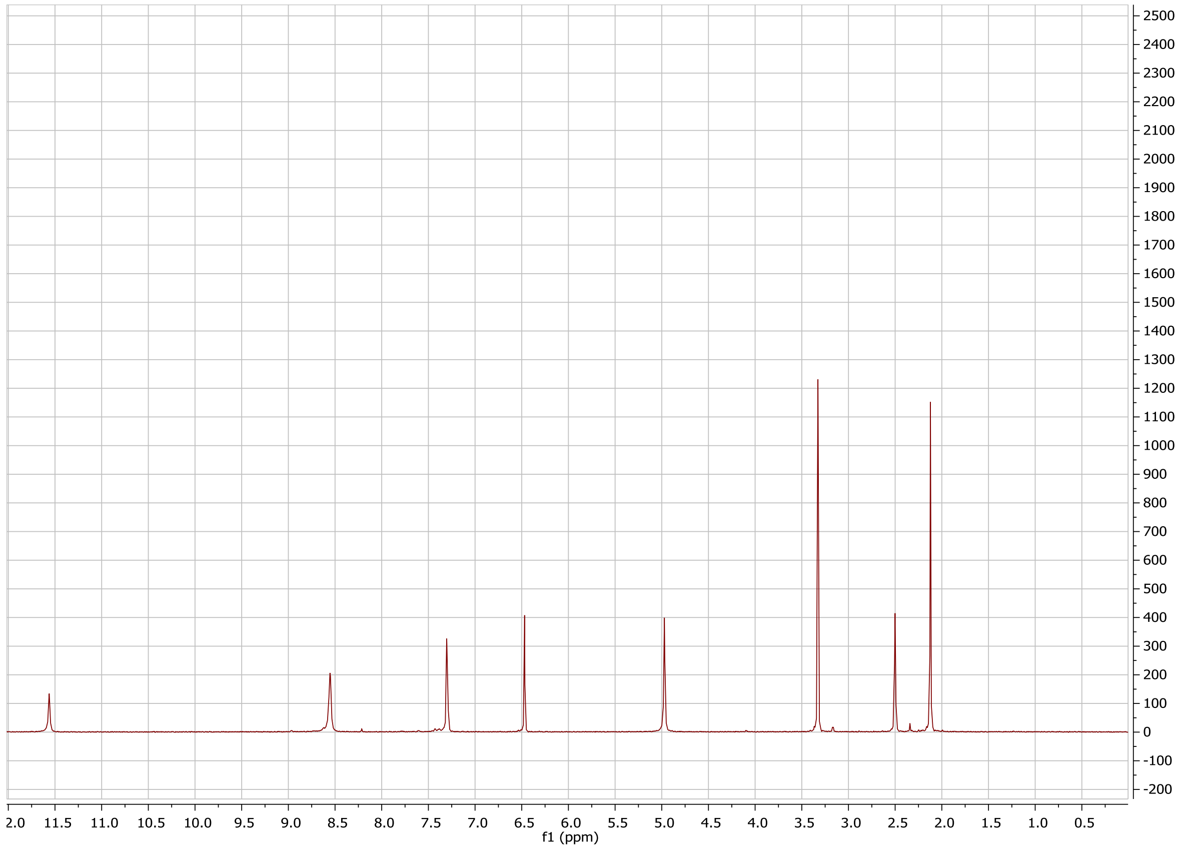
2500
2400
2300
2200
2100
2000
1900
1800
1700
1600
1500
1400
1300
1200
1100
1000
900
800
700
600
500
400
300
200
100
0
-100
-200
2.0 11.5 11.0 10.5 10.0 9.5 9.0 8.5 8.0 7.5 7.0 6.5 6.0 5.5 5.0 4.5 4.0 3.5 3.0 2.5 2.0 1.5 1.0 0.5
f1 (ppm)

Compound **16**

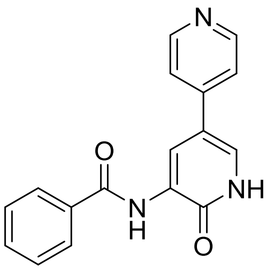
N
O
N
H
NH
O

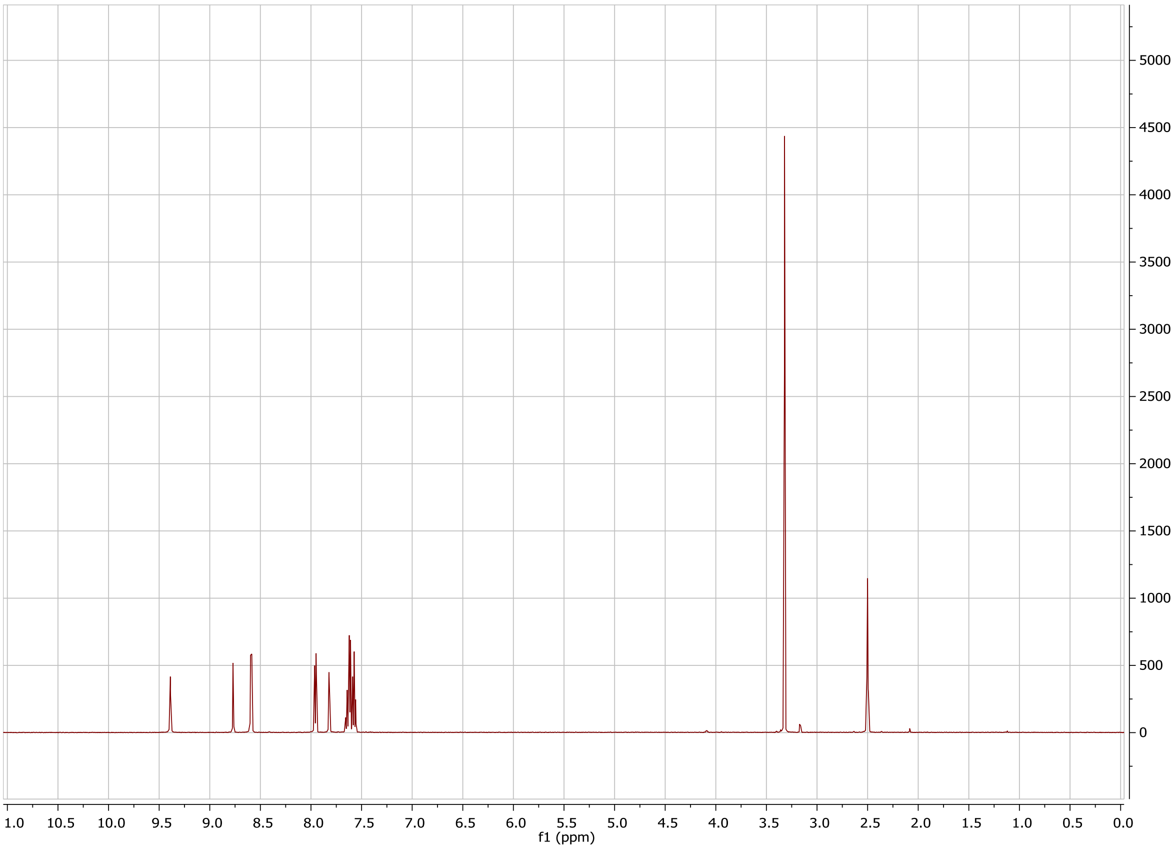
5000
4500
4000
3500
3000
2500
2000
1500
1000
500
0
1.0 10.5 10.0 9.5 9.0 8.5 8.0 7.5 7.0 6.5 6.0 5.5 5.0 4.5 4.0 3.5 3.0 2.5 2.0 1.5 1.0 0.5 0.0
f1 (ppm)

Compound **17**

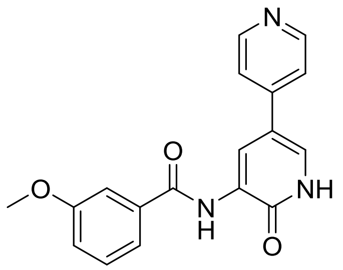

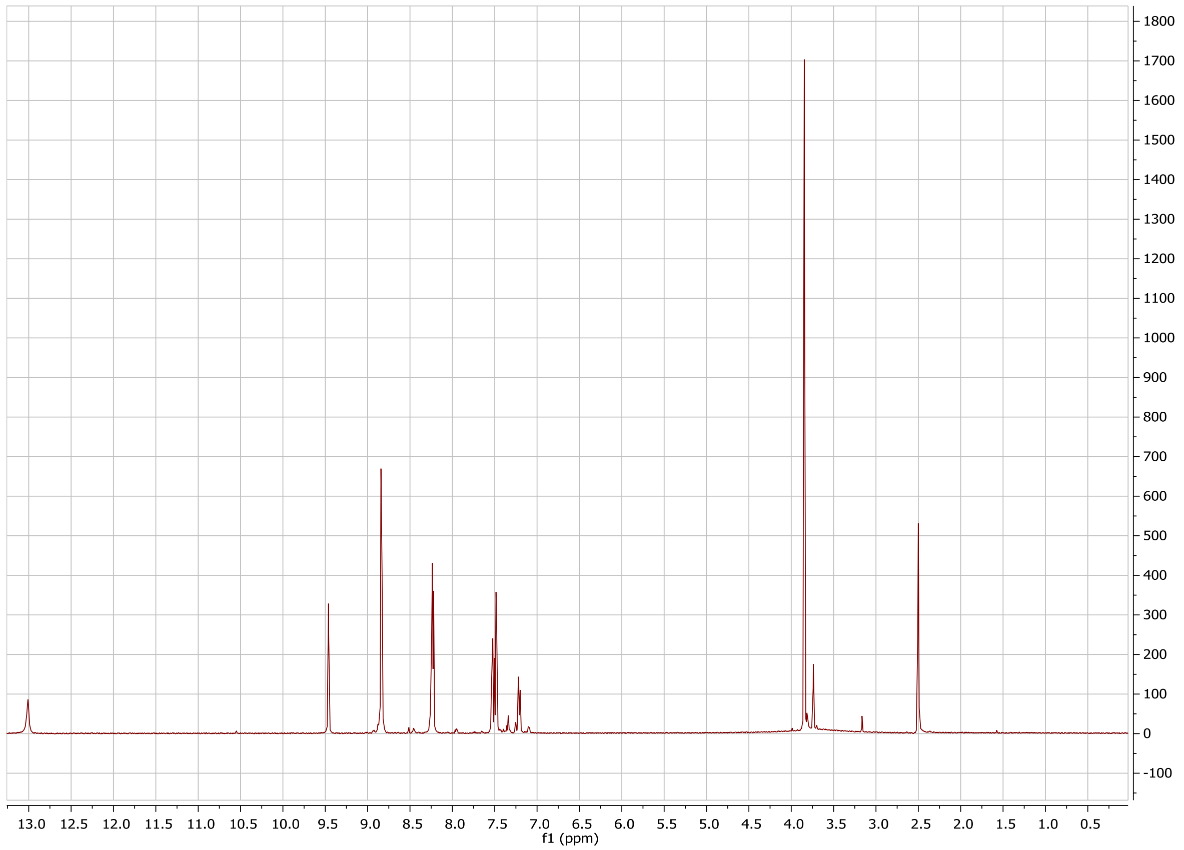

1800
1700
1600
1500
1400
1300
1200
1100
1000
900
800
700
600
500
400
300
200
100
0
-100
13.0 12.5 12.0 11.5 11.0 10.5 10.0 9.5 9.0 8.5 8.0 7.5 7.0 6.5 6.0 5.5 5.0 4.5 4.0 3.5 3.0 2.5 2.0 1.5 1.0 0.5
f1 (ppm)

Compound **18**

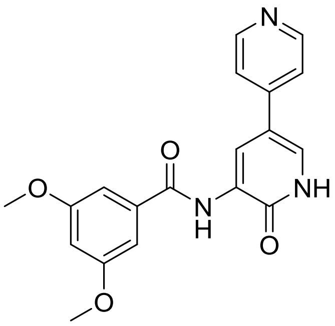
O
N
H
NH
O
O
N
O

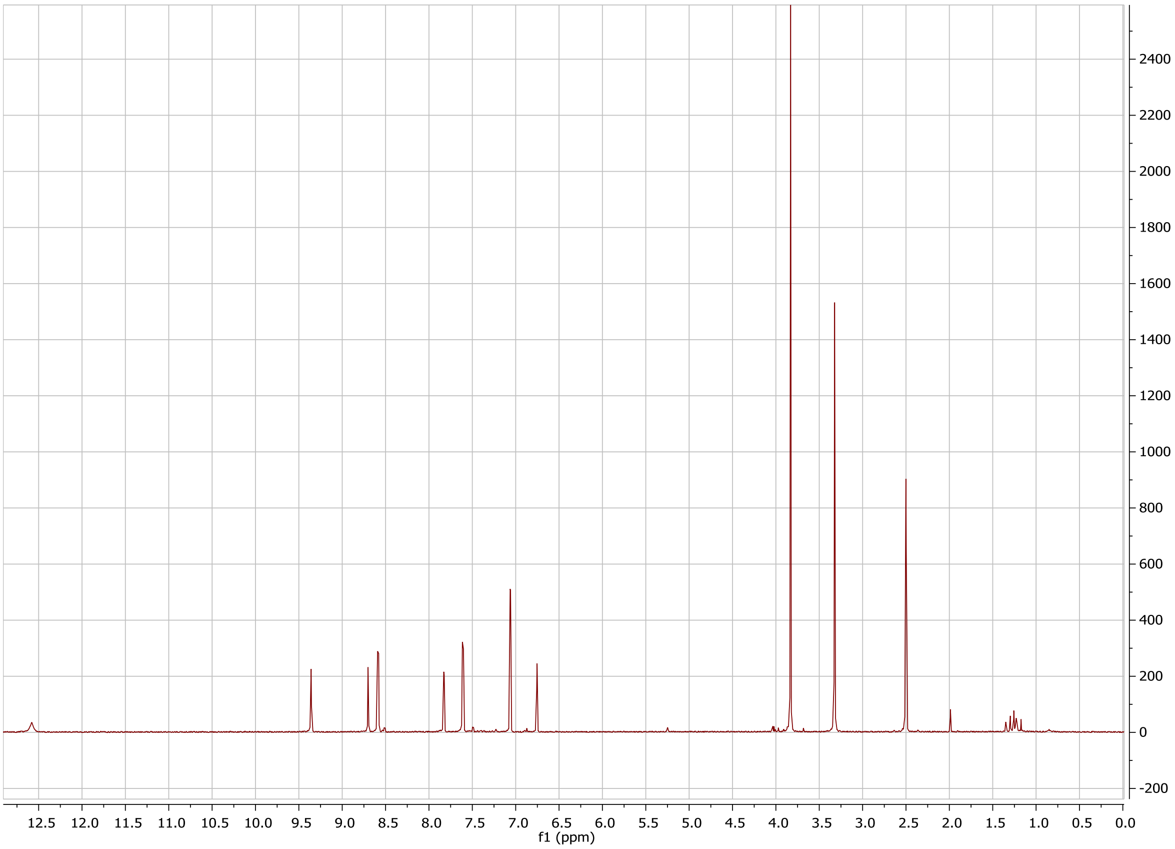
2400
2200
2000
1800
1600
1400
1200
1000
800
600
400
200
0
-200
12.5 12.0 11.5 11.0 10.5 10.0 9.5 9.0 8.5 8.0 7.5 7.0 6.5 6.0 5.5 5.0 4.5 4.0 3.5 3.0 2.5 2.0 1.5 1.0 0.5 0.0
f1 (ppm)

Compound **19**

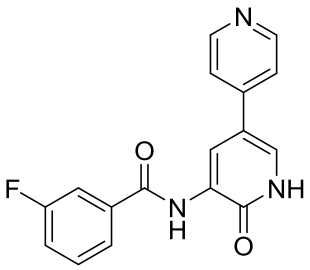
F
O
N
H
N
NH
O

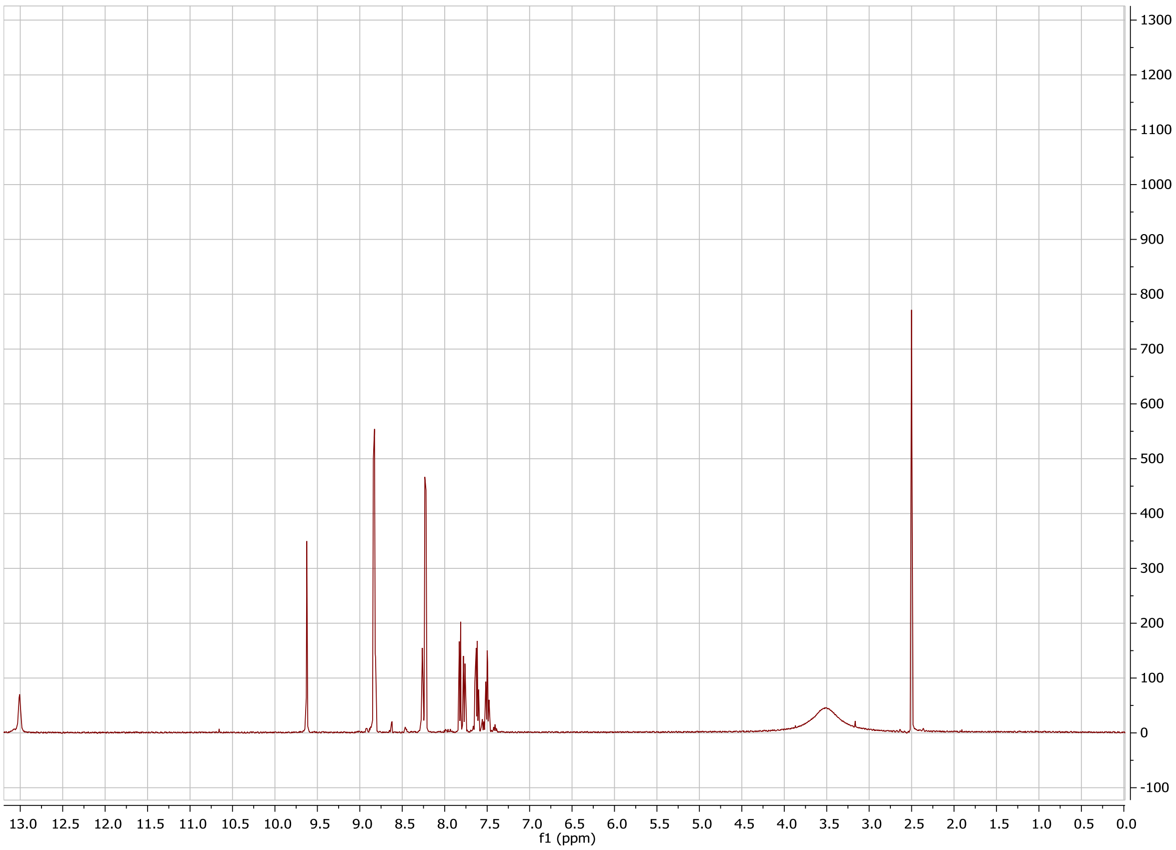
1300
1200
1100
1000
900
800
700
600
500
400
300
200
100
0
-100
13.0 12.5 12.0 11.5 11.0 10.5 10.0 9.5 9.0 8.5 8.0 7.5 7.0 6.5 6.0 5.5 5.0 4.5 4.0 3.5 3.0 2.5 2.0 1.5 1.0 0.5 0.0
f1 (ppm)

Compound **20**

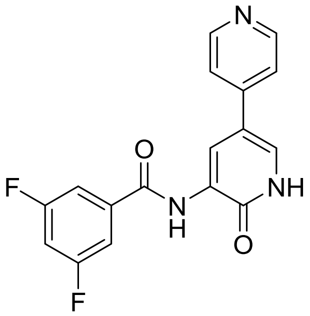

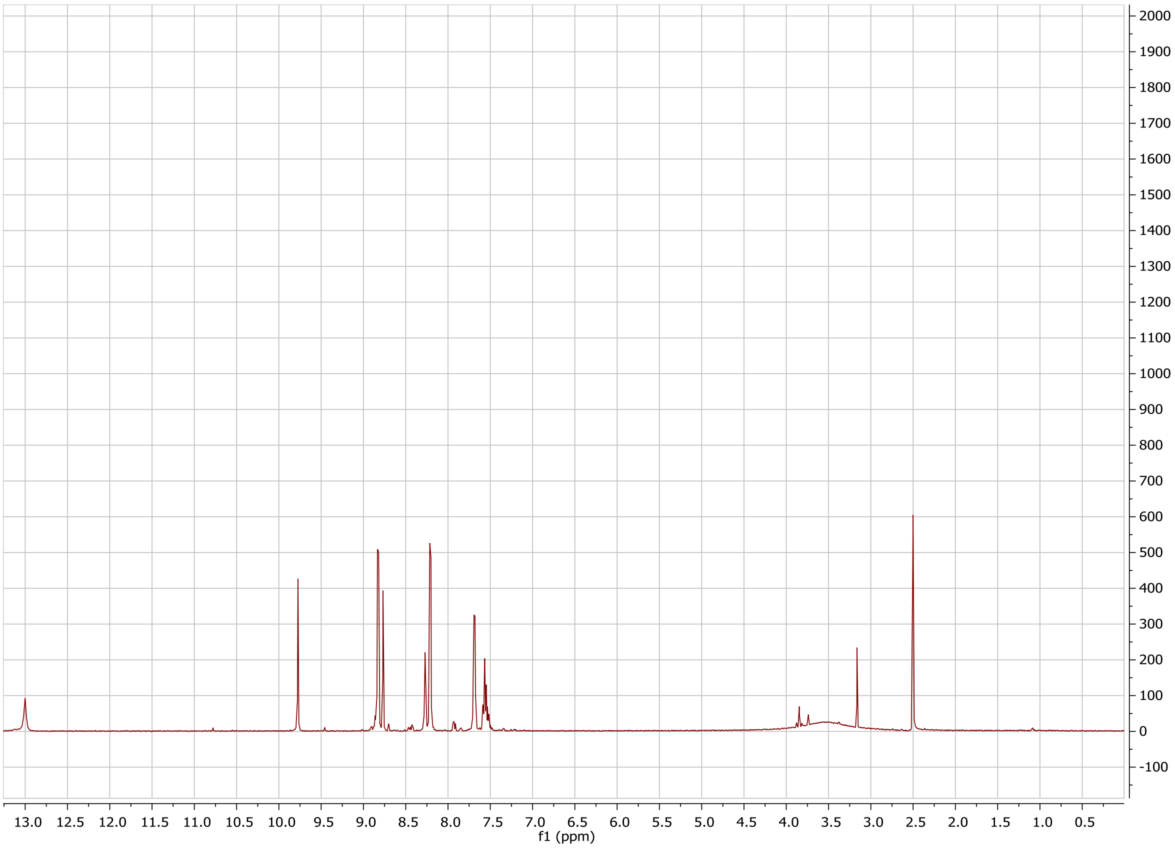

Compound **21**

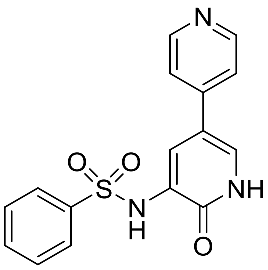

N
O
S
O
N
H
NH
O

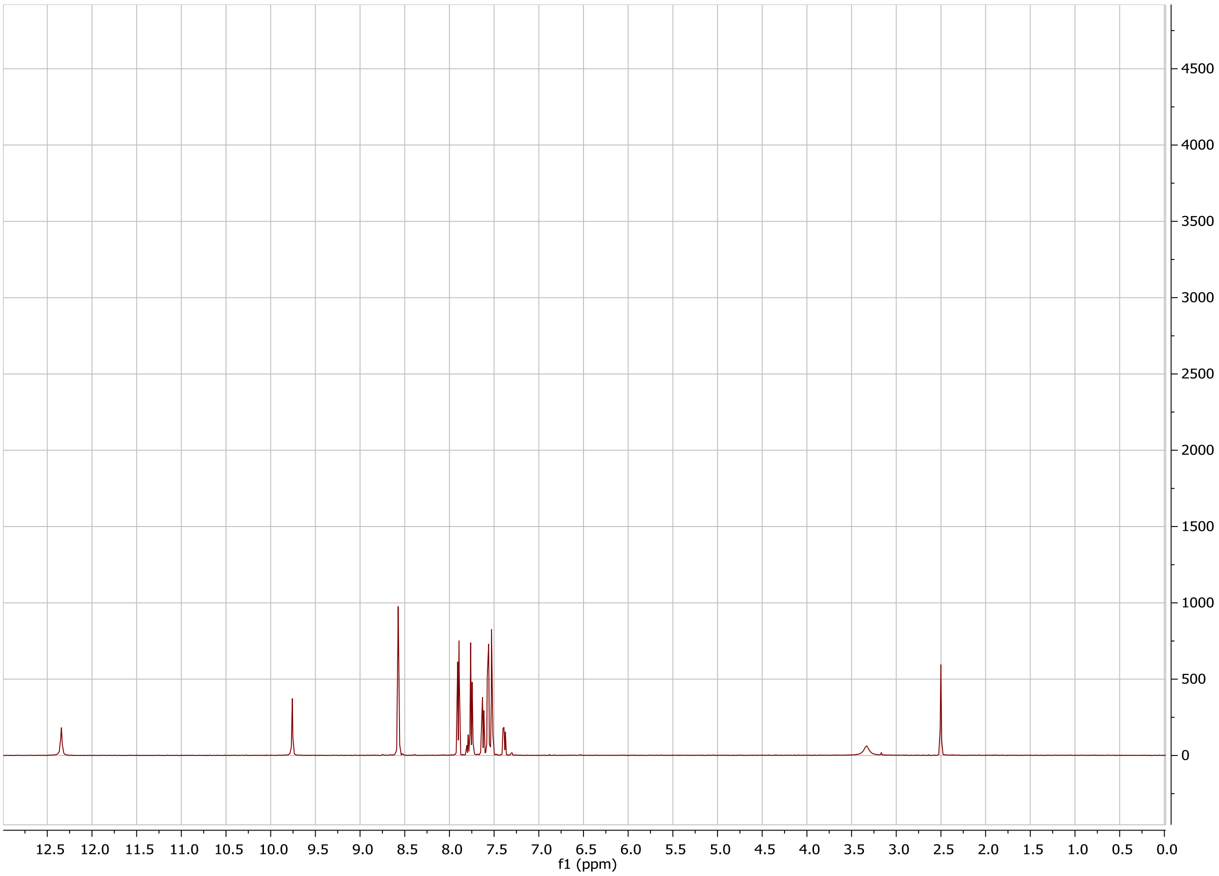

4500
4000
3500
3000
2500
2000
1500
1000
500
0
12.5 12.0 11.5 11.0 10.5 10.0 9.5 9.0 8.5 8.0 7.5 7.0 6.5 6.0 5.5 5.0 4.5 4.0 3.5 3.0 2.5 2.0 1.5 1.0 0.5 0.0
f1 (ppm)

Compound **22**

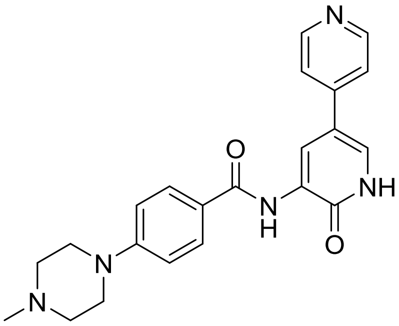

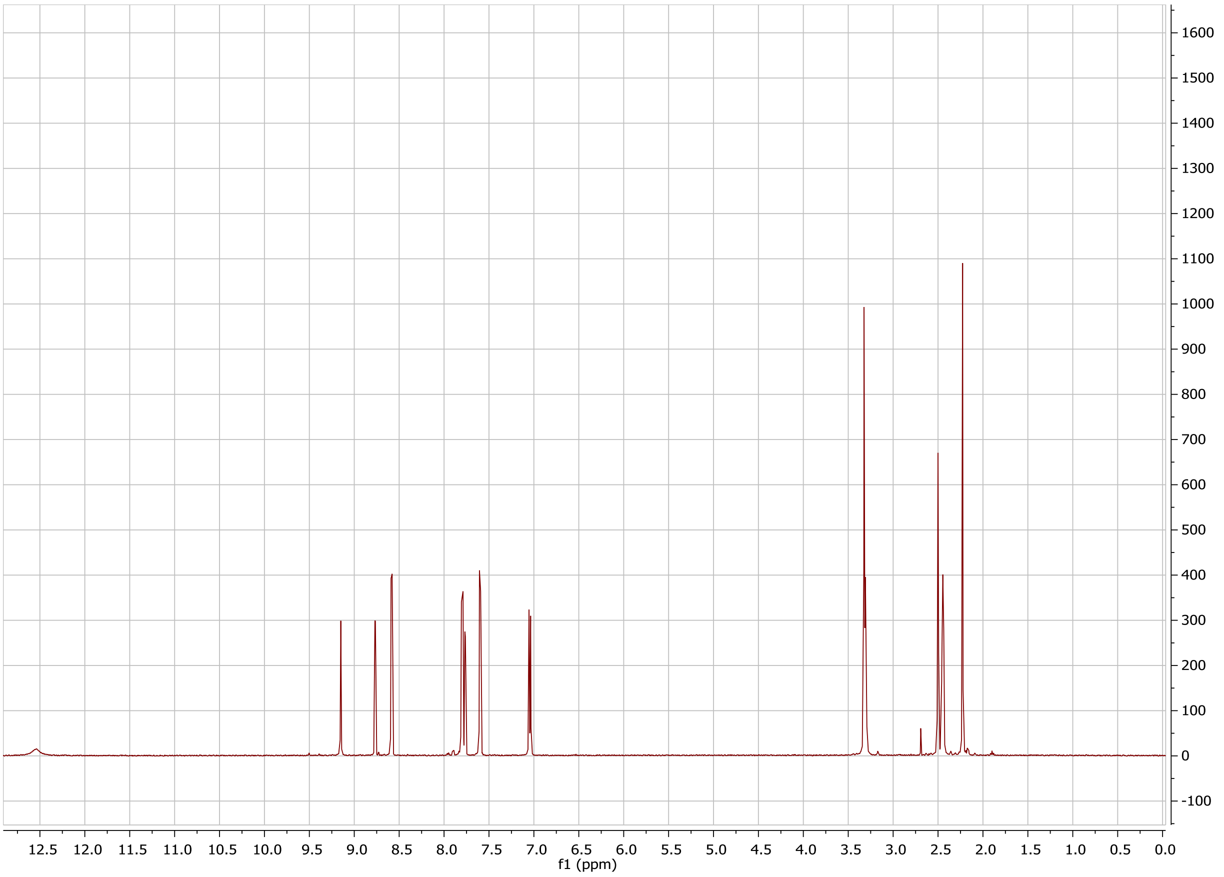

Compound **23**

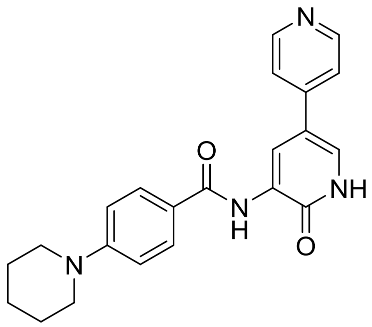

N
O
N
H
NH
O
N

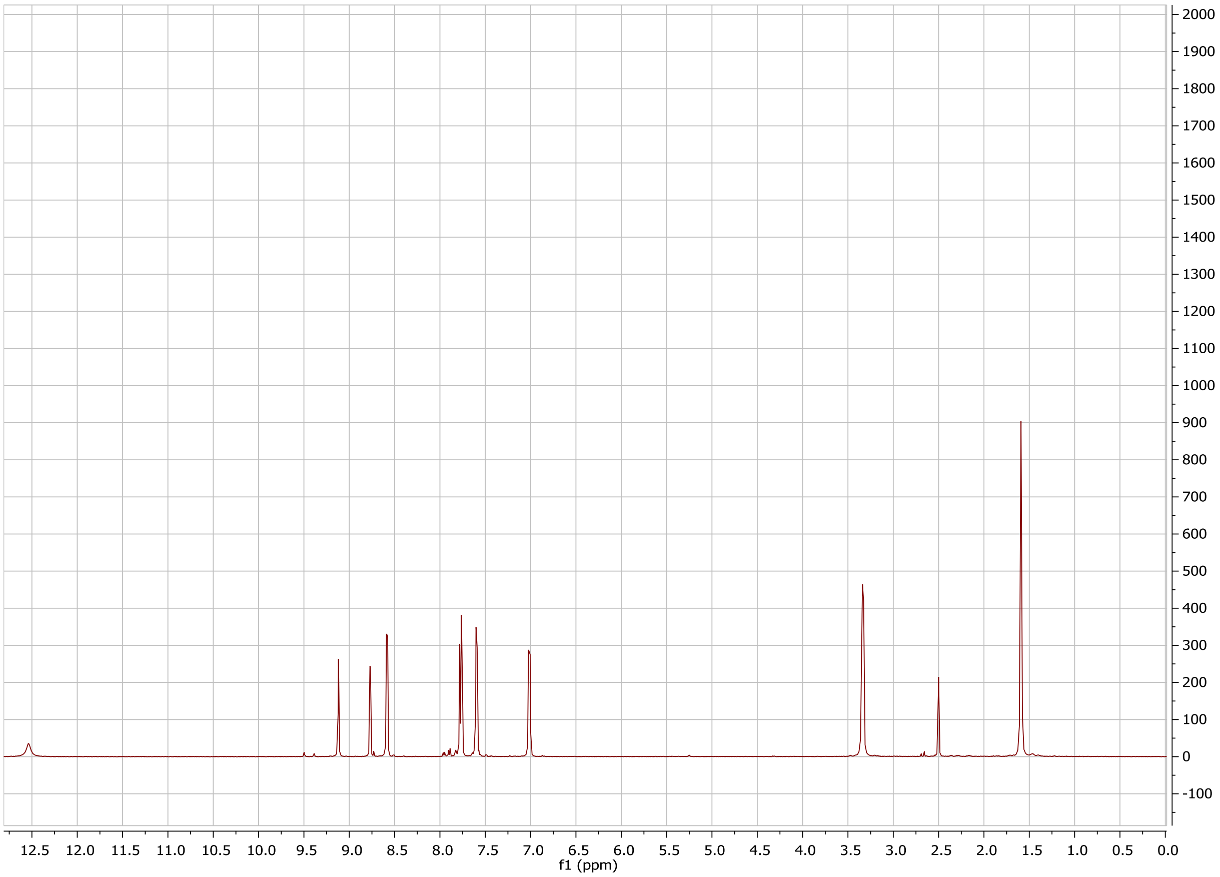

2000
1900
1800
1700
1600
1500
1400
1300
1200
1100
1000
900
800
700
600
500
400
300
200
100
0
-100
12.5 12.0 11.5 11.0 10.5 10.0 9.5 9.0 8.5 8.0 7.5 7.0 6.5 6.0 5.5 5.0 4.5 4.0 3.5 3.0 2.5 2.0 1.5 1.0 0.5 0.0
f1 (ppm)

Compound **24**

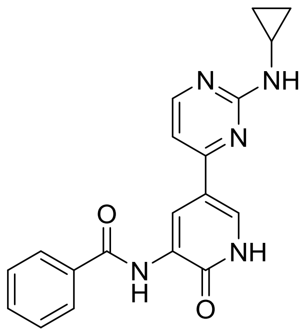
NH
N
N
O
N
H
NH
O

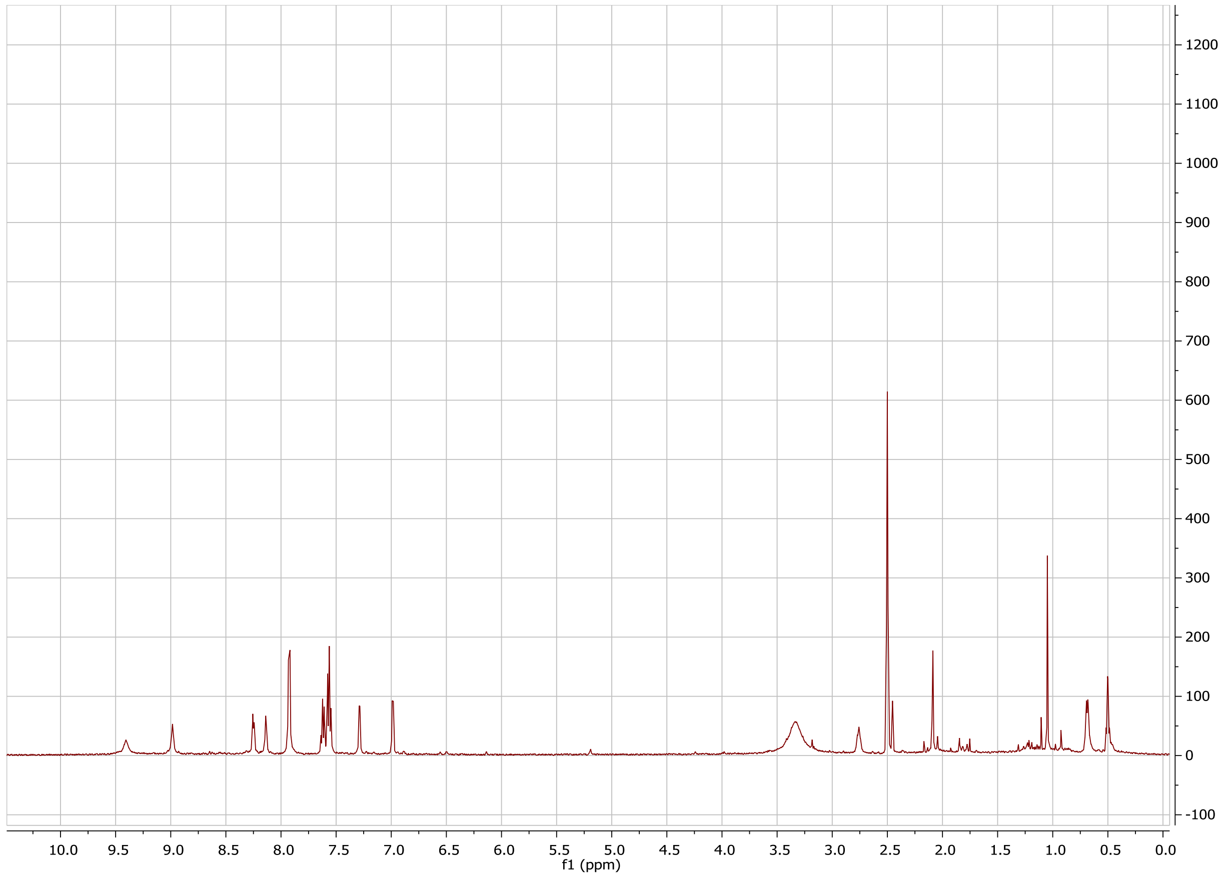
1200
1100
1000
900
800
700
600
500
400
300
200
100
0
-100
10.0 9.5 9.0 8.5 8.0 7.5 7.0 6.5 6.0 5.5 5.0 4.5 4.0 3.5 3.0 2.5 2.0 1.5 1.0 0.5 0.0
f1 (ppm)

References

[1] S. Naud, I. M. Westwood, A. Faisal, P. Sheldrake, V. Bavetsias, B. Atrash, K. M. J. Cheung, M. Liu, A. Hayes, J. Schmitt, A. Wood, V. Choi, K. Boxall, G. Mak, M. Gurden, M. Valenti, A. de Haven Brandon, A. Henley, R. Baker, C. McAndrew, B. Matijssen, R. Burke, S. Hoelder, S. A. Eccles, F. I. Raynaud, S. Linardopoulos, R. L. M. van Montfort, and J. Blagg. Structure-Based Design of Orally Bioavail-able 1*H*-Pyrrolo[3,2-*c*]pyridine Inhibitors of Mitotic Kinase Monopolar Spindle 1 (MPS1). Journal of Medicinal Chemistry, 56(24):10045–10065, 2013.

[2] R. J. Read, J. L Sussman, A. G. W. Leslie, and H. R. Powell. NATO Science Series, volume 245, pages 41–51. Springer Netherlands, 2007.

[3] M. D. Winn, C. C. Ballard, K. D. Cowtan, E. J. Dodson, P. Emsley, P. R. Evans, R. M. Keegan, E. B. Krissinel, A. G. W. Leslie, A. McCoy, S. J. McNicholas, G. N. Murshudov, N. S. Pannu, E. A. Potterton, H. R. Powell, R. J. Read, A. Vagin, and K. S. Wilson. Overview of the CCP4 suite and current develop-ments. Acta Crystallographica Section D, 67(4):235–242, Apr 2011.

[4] P. Evans. Scaling and assessment of data quality. Acta Crystallographica Section D, 62(1):72–82, Jan 2006.

[5] J. A. McCoy, R. W. Grosse-Kunstleve, P. D. Adams, M. D. Winn, L. C. Storoni, and R. J. Read. Phaser crystallographic software. Journal of Applied Crys-tallography, 40(4):658–674, Aug 2007.

[6] P. Emsley and K. Cowtan. Coot: model-building tools for molecular graphics. Acta Crystallographica Section D, 60(12 Part 1):2126–2132, Dec 2004.

[7] G. Bricogne, E. Blanc, M. Brandl, C. Flensburg, P. Keller, P. Paciorek, P. Roversi, A. Sharff, O. S. Smart, C. Vonrhein, and T. O. Womack. BUSTER, version 2.11.4. Global Phasing Ltd., Cambridge, United Kingdom, 2012.

[8] O. S. Smart, T. O. Womack, A. Sharff, C. Flensburg, P. Keller, W. Paciorek, C. Vonrhein, and G. Bricogne. Grade, version 1.2.1. Global Phasing Ltd.,

Cambridge, United Kingdom, 2012.

[9] I. J. Bruno, J. C. Cole, J. P. M. Lommerse, R. S. Rowland, R. Taylor, and M. L. Verdonk. Isostar: A library of information about nonbonded interac-tions. Journal of Computer-Aided Molecular Design, 11(6):525–537, 1997-11-01T00:00:00.

[10] I. W. Davis, A. Leaver-Fay, V. B. Chen, J. N. Block, G. J. Kapral, X. Wang, L. W. Murray, B. W. Arendall, J. Snoeyink, J. S. Richardson, and D. C. Richardson. Molprobity: all-atom contacts and structure validation for proteins and nucleic acids. Nucleic Acids Research, 35(suppl 2):W375–W383, 2007.

[11] P. A. Karplus and K. Diederichs. Linking crystallographic model and data quality. Science, 336(6084):1030–1033, 2012.
